# Design of sliding mode model predictive dual-loop control through self-learning strategy to mitigate the torque ripple in BLDC motor for electric vehicles

**DOI:** 10.1038/s41598-025-29445-w

**Published:** 2025-11-28

**Authors:** N. Prabhu, Thirumalaivasan Rajaram, Bragadeshwaran Ashok

**Affiliations:** 1 School of Electrical Engineering, Vellore Institute of Technology, Vellore, Tamilnadu 632014 India; 2 School of Mechanical Engineering, Vellore Institute of Technology, Vellore, Tamilnadu 632014 India

**Keywords:** BLDC motor, Electric vehicles, HIL and MIL simulation, Model predictive controller, Sliding mode controller, Energy science and technology, Engineering

## Abstract

This study investigates a novel dual-loop control strategy that combines sliding mode and model predictive controllers to reduce torque ripple in high-performance Brushless Direct Current (BLDC) motors, especially for automotive electric vehicle (EV) applications. The proposed control system merges the predictive features of Model Predictive Control (MPC) with the robustness of Sliding Mode Control (SMC), creating a dual-loop structure that optimizes inner-loop current regulation and outer-loop speed control. The cost function is formulated to regulate the d- and q-axis currents, enabling the calculation of the optimal output voltage signal necessary for efficient motor performance. This synergy ensures precise stator current modulation, effectively reducing torque ripple while maintaining superior motor efficiency and stability. Additionally, by incorporating adaptive heuristics and data-driven insights through a hybrid self-learning algorithm combining ANN and fuzzy logic, the SMC-MPC controller can forecast and reduce error rates in the BLDC motor, ensuring smooth torque output with minimal ripple. The performance of the SMC-MPC strategy is thoroughly evaluated through MATLAB/SIMULINK Model-in-the-Loop (MIL) simulations and validated via Hardware-in-the-Loop (HIL) testing. Comparative analysis shows that the proposed controller provides superior results, including a rapid 0.01 s rise time, a minimal 0.001% steady-state error, a 0.02 s settling time, and a peak overshoot of 0.066%, outperforming traditional PID and SMC controllers. Also, the experiments show a 28.57% reduction in torque ripple and efficiency maps, achieving 96.47% maximum efficiency. This endeavor validates that the SMC-MPC controller improves BLDC motor efficiency while extending the operational range of EVs.

## Introduction

**Fig. 1:**
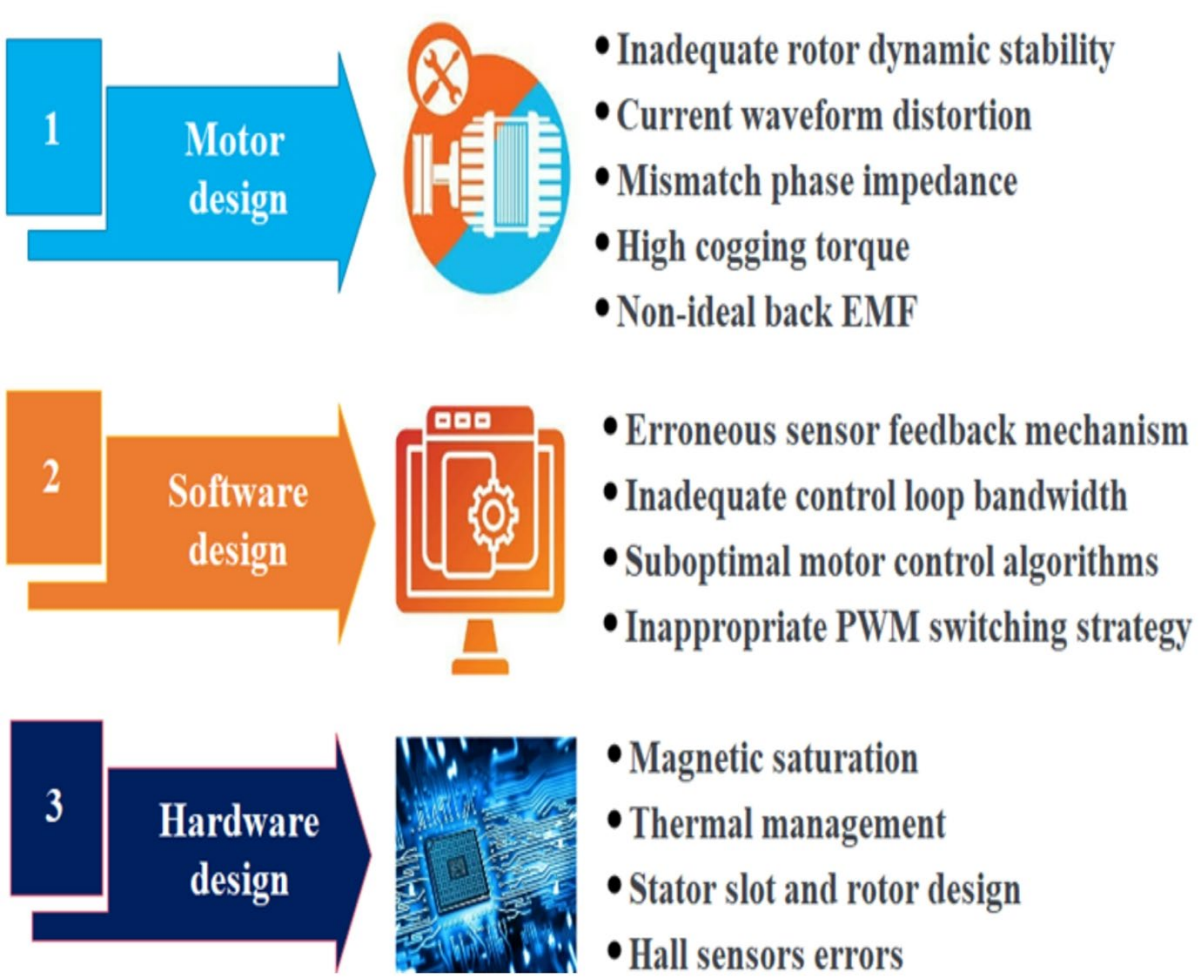
Causes of torque ripple for BLDC motor and controller design.

BLDC motors play a pivotal role in electric vehicles (EVs), offering efficient performance and consistent torque delivery. Valued for their reliability, efficiency, and safety, BLDC motors exhibit a linear relationship between torque, speed, and phase voltage, enhancing electronic commutation performance. Their compact structure, high efficiency, precise control capabilities, and excellent dynamic performance make them ideal for various applications, including EVs, automotive, and robotics^1,2^ Known as “electronically commutated motors,” BLDCs lack a commutator and brush setup, enhancing their suitability for demanding tasks with their design similar to permanent magnet synchronous motors. When a BLDC motor operates, torque is brought about by the precise synchronization of the magnetic fields from the permanent magnets with the stator windings. To achieve a smooth torque and velocity profile with low ripple, the operation of insulated gate bipolar transistors must be precisely synchronized with the rotor’s angular position, which is accurately found by the hall sensors^3,4^. While this stator current does not directly modulate initial torque production, meticulous control of torque ripple is vital to enhancing ride comfort and augmenting the operational efficiency of EVs. Contributing factors that intensify torque variation include velocity fluctuations, the intricate relationship among magnetomotive forces, and oscillations in air gap flux. This issue is particularly critical in motion-control applications for EV drives. To address these challenges, advanced control strategies have been developed. These methodologies aim to mitigate undesired torque component, maintain a consistent motor speed despite fluctuations in load and supply, and ensure optimal performance and energy efficiency for EVs. The quest for improved energy efficiency has driven the adoption of BLDC motors in EV applications, while simultaneously prompting advancements in electrical machine technology. Nevertheless, designing a BLDC motor controller involves a spectrum of sophisticated procedures, encompassing detailed mathematical modeling, advanced software design, and precise parameter refinement, all aimed at minimizing torque ripple^5,6^, as shown in Fig. 1.

Moreover, tuning these controllers requires computing the fundamental voltages the three-phase inverter needs to ensure optimal motor efficiency charcteristics at specific speeds. To minimise cyclic torque deviation, an Artificial Neural Network (ANN) incorporating a fuzzy self-adaptive algorithm, grounded in Sliding mode control and Model predictive control, is presently being devised. Field-Oriented Control (FOC) is a crucial control paradigm, predominantly utilized by BLDC-driven systems to achieve superior performance metrics^7^. This approach involves a dual closed-loop configuration, where the outer loop governs rotor speed to ensure exacting dynamic responses. In contrast, the inner loop modulates stator current, optimizing electromagnetic torque and minimizing distortions. Table 1 compares different torque ripple mitigation techniques for BLDC motors in EV applications.

**Table 1:** Comparative analysis of torque ripple control techniques in BLDC Motors.

Controller techniques used	Control techniques	Degree of torque ripple attenuation	Parameter optimization strategy	Challenges	Inference	References
PID	Linear	Low	Ziegler-Nichols tuning	Nonlinear dynamics and parametric uncertainties	Simple to implement, but may cause extended settling time and excessive peak	^8,9^
Fuzzy logic	Non-linear	Moderate	Fuzzy sets and rules-driven inference systems	Managing evolving conditions and time-intensive processes	Manage nonlinearities, though tuning fuzzy set functions and rules is complex	^12,13^
DTC	Non-linear	Moderate	Calibration of hysteresis bands	Fluctuations in switching frequency and inaccuracies in flux and torque prediction	Offers superior dynamics but with significant ripple and fluctuating switching frequency	^17,29^
FOC	Linear	Moderate	Optimizing PID controllers for both current and velocity control	Vulnerability to high-frequency noise and parameter	Exhibits steady-state performance, but precise parameter estimation is needed due to temperature variations	^18,29^
MPC	Non-linear	High	Forecasting of current response and formulation of the objective function	Implementation and computational complexity	Delivers superior torque oscillation suppression, enhanced predictive accuracy, and precise modeling	^19,43^
SMC	Non-linear	High	Derive the sliding mode surface and the control switching criteria	Oscillatory behavior	Robust against inconsistencies but prone to artifacts from measurement noise	^20,29^
SMC-MPC	Non-linear	High	Derivation of the sliding surface and synthesis of the cost function	Complexity integration and computational demands	Higher performance potential with higher computational requirements	^50–54^
ANN–FLC–SMC–MPC	Non-linear	High attenuation efficiency	ANN–FLC adaptively tunes SMC and simplifies MPC for fast, load-adaptive control	Real-time EV viability, hybrid tuning complexity, and high-quality ANN data	Highly precise, adaptive, and predictive capabilities for complex EV conditions	^55,56^

Although extensively utilized for motor current and speed regulation due to its inherent simplicity and robustness, the traditional PID (Proportional Integral Derivative) control strategy encounters profound challenges in high-performance drive systems^8,9^. These challenges primarily arise from constraints such as integral saturation and the intricate complexities of gain tuning amidst system uncertainties. Simulation analyses demonstrate that the PID controller exhibits significant inadequacies under load conditions, including impaired speed augmentation, excessive overshoot, pronounced oscillatory behavior, and extended settling times, undermining overall control efficacy. EV engineers have developed advanced control paradigms, like the Fuzzy Logic Controller (FLC), for BLDC motors^10,11^. The FLC is esteemed for its adeptness in mitigating torque ripple and enhancing dynamic and steady-state performance without necessitating an exact mathematical model. Compared to conventional PID controllers, FLC improves rise time, peak overshoot, and inrush current^12–14^. Nonetheless, FLC faces challenges such as handling rapid load disturbances, intricate rule-based design, and extended settling times. To counter these limitations, a hybrid PID-Fuzzy approach has been proposed, which dynamically adjusts PID gains to refine system response and stability^15,16^. Despite the advantages of PID and FLC, Direct Torque Control (DTC), Field-Oriented Control (FOC), Sliding Mode Controller (SMC), and Model Predictive Control (MPC) are frequently employed for their superior torque irregularity reduction and performance. DTC is renowned for its exceptional dynamic response but is burdened by torque ripple and electromagnetic interference (EMI)^17^. FOC offers precise control and stability but is susceptible to parameter variations and temperature fluctuations^18^. MPC delivers optimal control performance through predictive optimization and constraint handling over a defined prediction horizon, though it is limited by its high computational burden^19^. Each of these control strategies offers specific advantages and presents trade-offs, necessitating their selection based on application-specific requirements and performance criteria. Due to their capacity to manage the nonlinearities and uncertainties that are inherent in BLDC motor systems, Sliding Mode Controllers (SMCs) have gained a lot of attention in recent years. Specifically, when the load conditions are changeable, SMCs ensure improved control precision and superior torque voltage reduction. This is accomplished by driving the system states to a preset sliding range. But the continuing issue of chattering, which is characterized by high-frequency switching, remains a concern, despite mitigation efforts through higher-order and continuous sliding mode designs^20,21^. Recent studies have documented the integration of SMC and MPC as a significant advancement in the field of torque oscillation mitigation. This integration takes advantage of the complementary qualities of both techniques: SMC is robust against parameter fluctuations, external disturbances, and system uncertainties, whereas MPC optimizes control performance by forecasting future system behaviour and successfully managing constraints. The resulting SMC–MPC framework enhances overall system performance across a wide range of operating conditions, enabling proactive and precise torque control while maintaining resilience against external perturbations. Experimental testing regularly reveals that SMC-MPC outperforms traditional SMC and PID controllers in terms of ripple in the torque. and control precision, especially in the complex, real-time dynamics found in applications related to electric vehicles. Furthermore, the literature underscores the efficacy of this hybrid approach in reducing chattering, providing a more adaptive and predictive control solution^22–25^. Despite these developments, a substantial research gap remains regarding the incorporation of artificial intelligence methods, particularly Artificial Neural Networks (ANNs) and Fuzzy Logic Controllers (FLCs), within the SMC-MPC architecture. Although FLC and ANN have been applied separately in BLDC motor control, their combined performance and interaction within a unified SMC–MPC framework remain largely unexplored. Recent studies have demonstrated the potential of advanced MPC-based approaches in achieving effective torque ripple mitigation through optimized switching and duty-cycle adjustment. However, these methods exhibit a strong dependency on accurate motor modeling, leading to noticeable performance degradation under parameter uncertainties and transient disturbances. In parallel, enhanced direct torque control schemes incorporating composite controllers have shown improved torque and flux regulation; nevertheless, their adaptability remains constrained when subjected to nonlinear dynamic conditions. Furthermore, composite sliding mode control structures integrating differentiators and sliding mode observers have achieved superior dynamic tracking and reduced chattering, yet lack predictive adaptability and self-learning capabilities. In contrast, the proposed ANN–FLC–SMC–MPC hybrid control architecture synergistically combines the robustness of SMC, the predictive optimization of MPC, and the intelligent adaptability of ANN–FLC mechanisms. This unified configuration enables real-time adjustment of sliding surface parameters and predictive weights, resulting in minimized chattering, smoother transient performance, and substantial torque ripple reduction even under rapid load variations. Addressing this research void, the present work proposes a novel SMC-MPC-ANN-FLC control architecture for BLDC motor-driven EVs, filed as an Indian patent (Application No. 202541082634, August 31, 2025) to underscore its novelty and applicability.

In this proposed method, the ANN is used to improve the SMC component’s adaptability and resilience by learning optimal switching gains and adaptively adjusting the sliding surface parameters based on historical system behaviour and real-time error trend. Concurrently, the FLC is used to adapt control inputs through a rule-based mechanism, enabling effective handling of abrupt disturbances and optimizing the control effort. This intelligent integration not only minimizes the chattering commonly associated with traditional SMC systems, but it also reduces the computational complexity of the MPC by allowing approximate predictive modelling guided by ANN outputs. The suggested ANN-FLC-SMC-MPC framework performs well in both nominal and disturbed situations, with enhanced speed control, reduced torque fluctuations, and minimal steady-state error. In addition, Software-in-the-Loop (SIL) simulation results validate the proposed controller’s generalization capabilities, exhibiting stable and accurate performance across a broad range of driving situations with minimal estimation error and high convergence qualities. This controller provides a highly adaptive, forward-thinking, and computationally efficient solution designed exclusively for real-time use in electric vehicle drive systems to efficiently reduce torque fluctuations in BLDC motors.

## The current research article’s objective

Optimized torque regulation and ripple dampening in BLDC motors are pivotal for optimizing electric vehicle (EV) performance, thereby underscoring the necessity for sophisticated control mechanisms. Despite a comprehensive review of existing literature, which predominantly emphasizes the design of specialized controller modules, there remains a notable deficit in the thorough analysis of torque control strategies specifically tailored for BLDC motors in EV applications. Additionally, a significant gap exists in the critical examination of BLDC motor controller designs, with Hardware-in-the-Loop setups frequently overlooked and simulations primarily utilized for outcome assessment. To address these research deficiencies, this study seeks to rigorously assess current control approaches for proficiently minimizing torque oscillations in BLDC motors. The study employs an advanced methodology that synthesizes Model-in-the-Loop (MIL) and Hardware-in-the-Loop (HIL) simulations to assess and refine motor efficiency scrupulously. The experimental setup incorporates a 3 kW, three-phase BLDC motor embedded in the HIL architecture, connected via a data acquisition system and real-time analytical tools to rigorously assess a range of controllers developed using MATLAB/Simulink. The approach optimizes Sliding Mode-Model Predictive Control (SMC-MPC) parameters using Artificial Neural Networks (ANNs) coupled with a fuzzy self-learning algorithm. The current implementation focuses primarily on conventional system dynamics, with the SMC framework being inherently resistant to a class of matched uncertainty due to its discontinuous control action. The primary objective of this research is to demonstrate the feasibility and usefulness of SMC in achieving steady tracking and control under different load conditions as a reference. Further, the inner-loop MPC design uses a modified approach to reduce computing load while keeping the vital characteristics of predictive control, which, though limiting some aspects of typical MPC performance, provides real-time applicability and is appropriate for embedded control instances. Moreover, fuzzy logic and artificial neural network (ANN) based techniques are employed as function approximators to achieve an optimal balance among control granularity and computing efficiency, especially in applications requiring rapid execution and real-time response. This refined methodology is designed to enhance motor efficiency, address stringent vehicular energy and range requirements, and effectively reduce speed and oscillations in torque across varied loading conditions, thereby markedly elevating the performance of BLDC motors in EVs.

Overall, “Experimental methodology” outlines the experimental methodology, including MIL and HIL validation setups. “Mathematical development and design of the BLDC motor controller” presents the mathematical modeling and controller design. “Development of a proposed SMC-MPC controller for mitigating torque ripplein BLDC motors” details the proposed dual-loop SMC–MPC control with ANN–FLC adaptation. “MIL Simulation-based comparative analysis of the SMC-MPC controllermethod and conventionalcontrollers” provides the MIL-based comparative analysis with conventional controllers. Finally, “Development of BLDC Motor controllers using HIL simulation” validates the proposed approach through HIL simulation and real-time efficiency evaluation.

## Experimental methodology

**Fig. 2:**
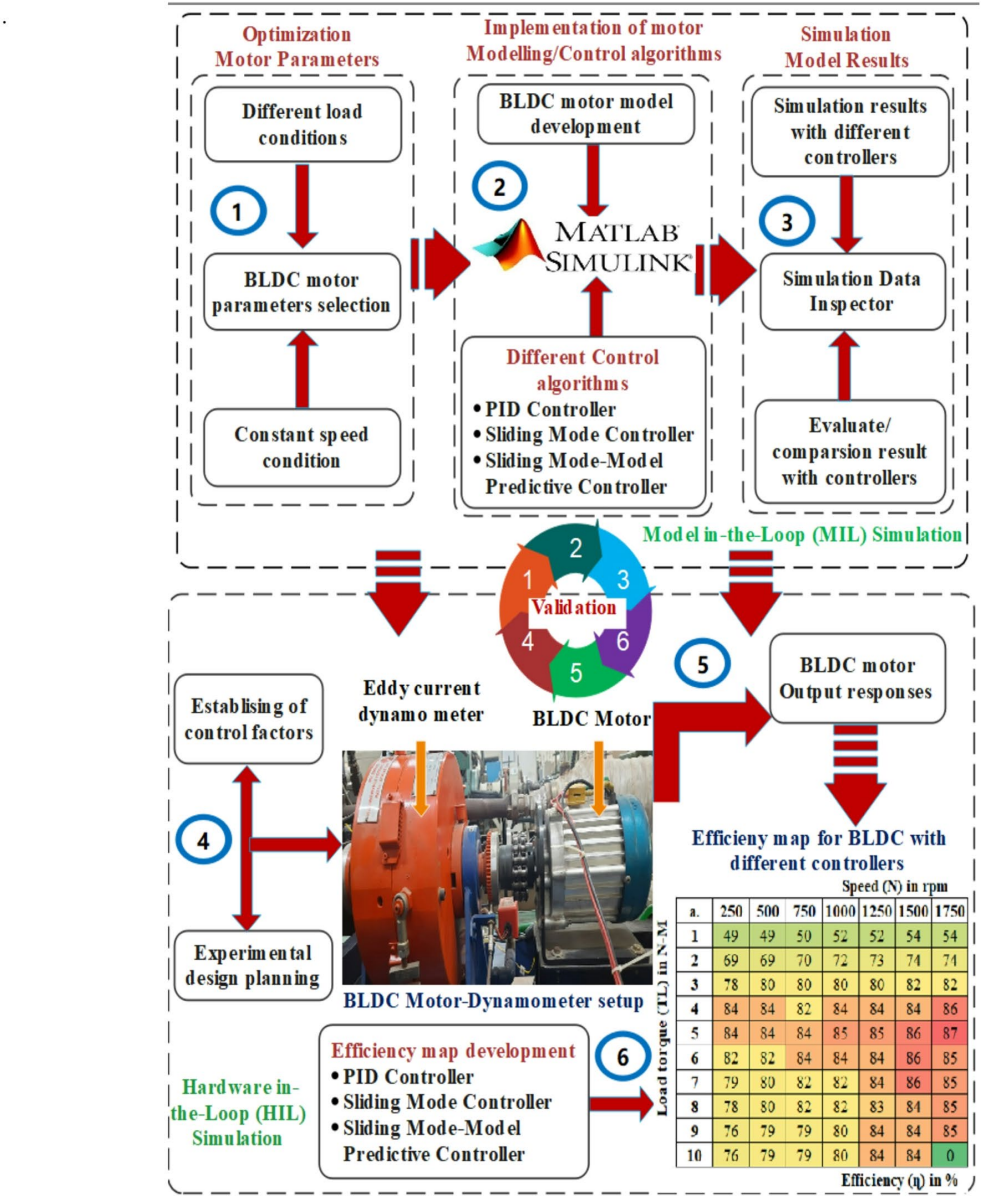
Investigational methodology for Torque ripple control of BLDC motor in EVs.

The proposed methodology explains the behavior of speed and torque regulation, as well as the performance metrics of BLDC motors across different control methods for EVs. Using MIL and HIL simulations, torque fluctuation control is examined with PID, sliding mode, and model predictive control. Fig 2 shows the research strategy and execution. To ensure reproducibility, key experimental parameters are clearly defined and documented as follows. The 100 µs sampling interval matches the inverter switching cycle, reducing interference effects and enabling accurate current control. The 1.2 kHz filter bandwidth effectively balances noise reduction with real-time responsiveness in the feedback loop. Load transition tests are conducted under load conditions from 3 N m to 6 N m at 3000 rpm, covering both steady-state and transient operating conditions. These settings provide consistent evaluation of torque ripple, speed regulation, and dynamic response across all control strategies. The HIL platform was implemented using the proposed hybrid SMC–MPC real-time controller platform, which integrates ANN–FLC-based adaptive tuning, real-time PWM generation, and torque feedback synchronization. This setup enables precise evaluation of controller robustness and torque ripple suppression. Each test was repeated three times under identical conditions, and the averaged results were considered to ensure measurement consistency and repeatability.

### Model-in-the-loop (MIL) evaluation

The elaborate integration of a two-level three-phase inverter, controller, and BLDC motor design in MIL evaluation facilitates the refinement and sophistication of controller algorithms for highly complex experimental simulations. Simulink is used to develop controller models for desktop simulations, along with the SimScape Electrical toolbox. A three-phase inverter, equipped with six IGBT switches, drives the BLDC motor, employing precise commutation and switching sequence logic to ensure seamless operation. Initially, BLDC motor drive behaviour is optimized through parameter tuning based on varying load conditions while maintaining a constant speed, as detailed in Table 2. The mathematical model of a BLDC motor is articulated in MATLAB, incorporating control systems, switching mechanisms, sensors, and a suite of ancillary components. Torque ripple effects produced by parameter fluctuations and load disturbances are minimized using control strategies (PID, sliding mode, and sliding mode -model predictive controller). The simulation results assess time response and system performance under varying loads, evaluated and compared with an advanced data analyzer.

### Hardware-in-the-loop (HIL) evaluation

Following the MIL simulation, controller outcomes are verified through HIL simulation. The HIL model architecture, developed using advanced experimental design, provides a detailed evaluation framework for operating a BLDC motor under different control scenarios. Through Hardware-in-the-Loop evaluation, the BLDC motor’s control system is exhaustively tested. The system model leverages the model-based calibration toolbox strategy for deciding how inputs affect outputs. HIL systems for BLDC motor drive controllers are gaining prominence in EVs, requiring enhanced dynamic response and high-fidelity models to reduce ripples in torque by optimizing controller parameters. The BLDC motor is scrutinized within precise tolerance boundaries, with rigorously measured parameters including stator current, torque, speed, and terminal conditions. Using advanced controllers, design strategies are rigorously evaluated under varying loads to enhance precision and control ripples present in the torque. Controller output responses establish torque fluctuations analysis and BLDC motor drive performance. Electrical efficiency contour maps reveal energy conversion performance across various EV operating points, identifying the optimal speed-torque relationship for BLDC drives.

**Table 2:** Control and experimental parameter ranges for evaluation.

Control variables	Category	Minimum	Maximum	Units
Speed	Quantitative	250	3000	Rpm
Load	Quantitative	0	10	N-m

## Mathematical development and design of the BLDC motor controller

**Fig. 3:**
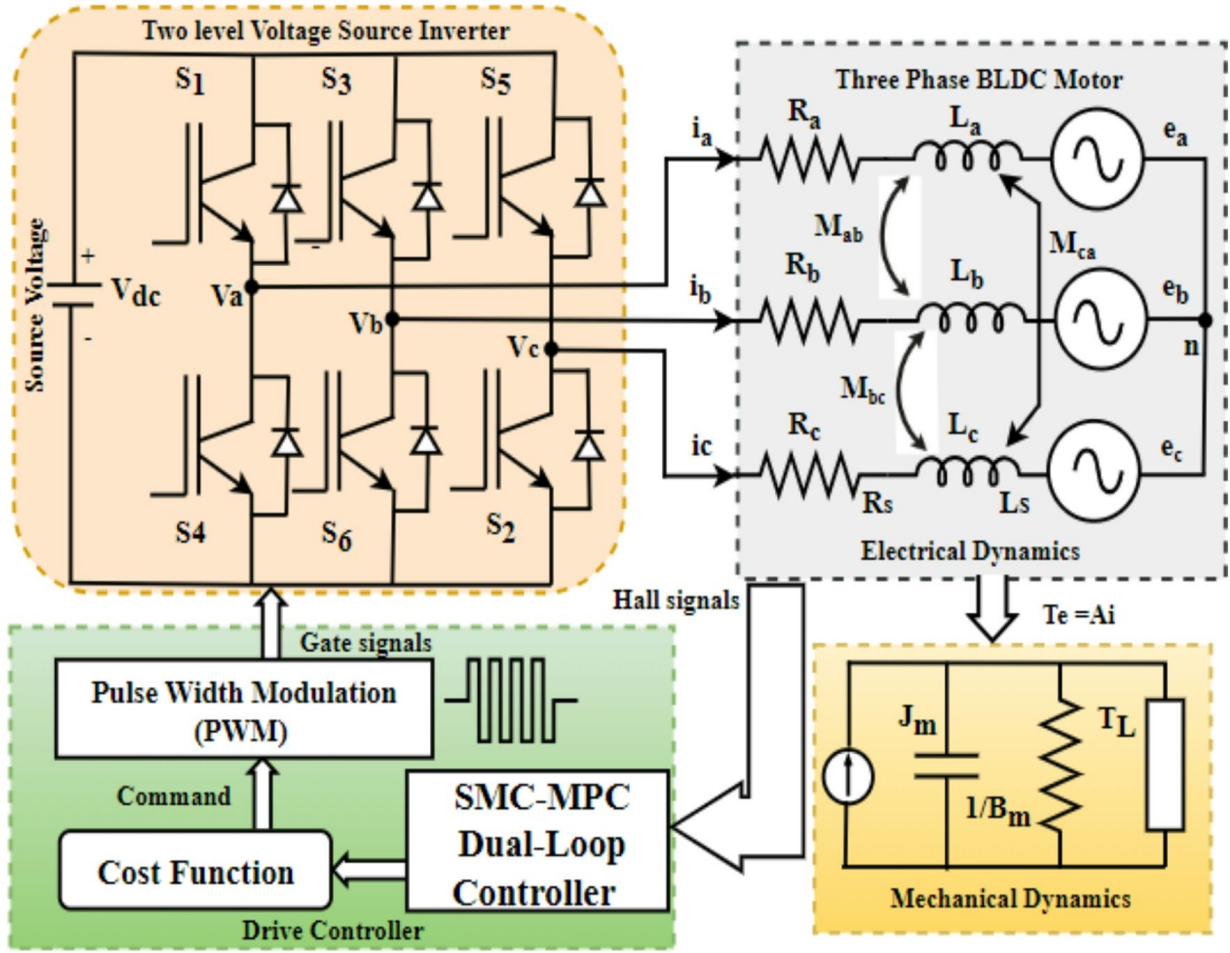
Mathematical model of sliding mode model predictive controller fed BLDC motor.

Figure 3 illustrates an analytical formulation of the BLDC motor model. The model integrates a DC voltage source, a two-level three-phase inverter, and a BLDC motor with star-connected three-phase stator windings, while its rotor comprises permanent magnet assemblies. Each motor phase is electrically modeled by a series circuit with inductance L_s_, mutual inductances M_s_, resistance R_s_, and trapezoidal back EMF signals e_a_​, e_b_​, and e_c_​, evaluation of the phase voltage measured from the neutral reference point V_n_. The dynamics of the three-phase stator windings in a BLDC motor are governed by Eqs. (1, 2, and 3).

1 $$\:{\mathrm{V}}_{\mathrm{a}}\:=\:{\mathrm{R}}_{\mathrm{a}}\:{\mathrm{i}}_{\mathrm{a}\:}+\left(\mathrm{L}-\mathrm{M}\right)\:\frac{\mathrm{d}}{\mathrm{d}\mathrm{t}}{\mathrm{i}}_{\mathrm{a}}+{\mathrm{e}}_{\mathrm{a}}+\mathrm{V}\mathrm{n}$$

2 $$\:{\mathrm{V}}_{\mathrm{b}}\:=\:{\mathrm{R}}_{\mathrm{b}}\:{\mathrm{i}}_{\mathrm{b}\:}+\left(\mathrm{L}-\mathrm{M}\right)\:\frac{\mathrm{d}}{\mathrm{d}\mathrm{t}}{\mathrm{i}}_{\mathrm{b}}+{\mathrm{e}}_{\mathrm{b}}+\mathrm{V}\mathrm{n}$$

3 $$\:{\mathrm{V}}_{\mathrm{c}}\:=\:{\mathrm{R}}_{\mathrm{c}}\:{\mathrm{i}}_{\mathrm{c}}+\left(\mathrm{L}-\mathrm{M}\right)\:\frac{\mathrm{d}}{\mathrm{d}\mathrm{t}}{\mathrm{i}}_{\mathrm{c}}+{\mathrm{e}}_{\mathrm{c}}+\mathrm{V}\mathrm{n}$$

4 $$\:{\mathrm{R}}_{\mathrm{a}}=\:{\mathrm{R}}_{\mathrm{b}}={\mathrm{R}}_{\mathrm{c}}=\mathrm{R}\mathrm{s}$$

5 $$\:{\mathrm{L}}_{\mathrm{a}}=\:{\mathrm{L}}_{\mathrm{b}}={\mathrm{L}}_{\mathrm{c}}=\mathrm{L}$$

6 $$\:{\mathrm{M}}_{\mathrm{a}\mathrm{b}}={\mathrm{M}}_{\mathrm{b}\mathrm{a}}={\mathrm{M}}_{\mathrm{a}\mathrm{c}}={\mathrm{M}}_{\mathrm{c}\mathrm{a}}={\mathrm{M}}_{\mathrm{b}\mathrm{c}}={\mathrm{M}}_{\mathrm{c}\mathrm{b}}=\mathrm{M}$$

7 $$\:\mathrm{L}-\mathrm{M}=\:\mathrm{L}\mathrm{s}$$

where V_a_, V_b_, and V_c_ represent the phase voltages; e_a_, e_b_, and e_c_ denote the back electromotive forces, i_a_, i_b_, and i_c_ are phase currents, R_a_, R_b_, and R_c_ represent the phase resistances, R_s_ signifies the equivalent stator resistance, L_a_, L_b_, and L_c_ are the self-inductances of each phase, L_s_ = L - M is the equivalent phase inductance, while the mutual inductances are specified as M_ab_, M_ca._, and M_bc_ by the following Eqs. (4, 5, 6 and 7). Additionally, the connection of the stator winding must be constrained to ensure balance in Eqs. (8),

8 $$\:{\mathrm{i}}_{\mathrm{a}}+\:{\mathrm{i}}_{\mathrm{b}}\:+\:{\mathrm{i}}_{\mathrm{c}}\:=\:0$$

In the three-phase (abc) stator frame of reference, motor voltage and current oscillate even under constant speed and load, rendering them inadequate for control law equations. However, to maintain constant torque, both the currents and voltages within the d-q reference framework must be equivalent^26–28^. To effectuate this transformation, employ the Clarke and Park transformations to convert from abc to d-q coordinates, and apply the d-q reference frame phase voltage Eqs. (9) and (10) for the BLDC motor as follows,

9 $$\:{\mathrm{V}}_{\mathrm{s}\mathrm{d}}\:=\:{\mathrm{R}}_{\mathrm{s}}\:{\mathrm{i}}_{\mathrm{d}}+\mathrm{L}\mathrm{s}\mathrm{d}\:\:\frac{\mathrm{d}}{\mathrm{d}\mathrm{t}}{\mathrm{i}}_{\mathrm{d}}-{\upomega\:}\mathrm{e}\:\mathrm{L}\mathrm{s}\mathrm{q}\:\mathrm{i}\mathrm{q}$$

10 $$\:{\mathrm{V}}_{\mathrm{s}\mathrm{q}}\:=\:{\mathrm{R}}_{\mathrm{s}}\:{\mathrm{i}}_{\mathrm{q}}+\mathrm{L}\mathrm{s}\mathrm{q}\:\:\frac{\mathrm{d}}{\mathrm{d}\mathrm{t}}{\mathrm{i}}_{\mathrm{q}}+{\upomega\:}\mathrm{e}\:\mathrm{L}\mathrm{s}\mathrm{d}\:\mathrm{i}\mathrm{d}\:+{\upomega\:}\mathrm{e}\:{\uppsi\:}_r$$

From Eqs. (9) and (10), the state Eqs. (11) and (12) of the BLDC motor in the d-q reference frame are as follows,

11 $$\:\frac{\mathrm{d}}{\mathrm{d}\mathrm{t}}{\mathrm{i}}_{\mathrm{d}}=\:\frac{-\mathrm{R}\mathrm{s}}{\mathrm{L}\mathrm{s}\mathrm{d}}{\mathrm{i}}_{\mathrm{d}}+{\upomega\:}\mathrm{e}\:\frac{\mathrm{L}\mathrm{s}\mathrm{q}}{\mathrm{L}\mathrm{s}\mathrm{d}}{\mathrm{i}}_{\mathrm{q}}+\:\frac{\mathrm{V}\mathrm{s}\mathrm{d}}{\mathrm{L}\mathrm{s}\mathrm{d}}$$

12 $$\:\frac{\mathrm{d}}{\mathrm{d}\mathrm{t}}{\mathrm{i}}_{\mathrm{q}}=\:\frac{-\mathrm{R}\mathrm{s}}{\mathrm{L}\mathrm{s}\mathrm{q}}{\mathrm{i}}_{\mathrm{q}}-{\upomega\:}\mathrm{e}\:\frac{\mathrm{L}\mathrm{s}\mathrm{d}}{\mathrm{L}\mathrm{s}\mathrm{q}}{\mathrm{i}}_{\mathrm{d}}+\:\frac{\mathrm{V}\mathrm{s}\mathrm{q}}{\mathrm{L}\mathrm{s}\mathrm{q}}\:-{\upomega\:}\mathrm{e}\:\frac{{\uppsi\:}\mathrm{r}\:}{\mathrm{L}\mathrm{s}\mathrm{q}}$$

where V_sd_ and V_sq_ represent the phase voltage components along the d axis and q axis, i_d_ and i_q_ denote the phase current components along the d axis and q axis, L_sd_ and L_sq_ indicate the stator inductance components in the d axis and q axis, ψ_r_ refers to the rotor flux linkage, and ω_e_ represents the electrical angular velocity.

The electromagnetic torque of a BLDC motor is expressed by Eq. (13) in the d−q reference frame as follows,

13 $$\:\mathrm{T}\mathrm{e}=1.5\:\mathrm{P}\:[{\uppsi\:}\mathrm{r}+(\mathrm{L}\mathrm{s}\mathrm{d}-\:\mathrm{L}\mathrm{s}\mathrm{q}\left)\:\mathrm{i}\mathrm{d}\right]\:\mathrm{i}\mathrm{q}\:$$

By adopting the control approach i_d_​=0, Eq. (13) is simplified to Eq. (14),

14 $$\:\mathrm{T}\mathrm{e}=1.5\:\mathrm{P}\:{\uppsi\:}\mathrm{r}\:\mathrm{i}\mathrm{q}\:\:\:\:$$

where T_e_ represents the electromagnetic torque and P denotes the pole pairs count.

The mechanical equation governing the BLDC motor is given by Eq. (15).

15 $$\:\dot{{{\upomega\:}}_{\mathrm{e}}}=\:\frac{\mathrm{P}\:}{\mathrm{J}\mathrm{m}}\:(\mathrm{T}\mathrm{e}-\mathrm{T}\mathrm{L}\:-\mathrm{B}\mathrm{m}{\upomega\:}\mathrm{m})\:\:$$

where T_L_​ denotes the load torque, B_m​_ is the damping constant, J_m_ represents the rotational inertia, and B_m_​ω_m_​ signifies the viscous damping torque.

The key specifications for the BLDC motor used in the electric vehicle are outlined in Table 3.

**Table 3:** Specifications of brushless DC motor drive systems.

SNO	Specifications	Values
1	Supply DC voltage	60 V
2	Rated motor current	65 A
3	Rated rotor speed	3000 Rpm
4	Rated motor power	3KW
5	Rated motor torque	10 N-m
6	Phase inductance	0.0085 H
7	Phase resistance	2.875 Ohms
8	No.of pole pairs	3
9	Type of winding	Star wound
10	Friction coefficient	0.045 kg/ms
11	Inertia constant	0.08 kg. m^2^
12	Torque constant	1.5 N-m/A
13	Back EMF constant	1.3 V/rad/s
14	Rotor initial position	0^o^ Degree

## Development of a proposed SMC-MPC controller for mitigating torque ripple in BLDC motors

**Fig. 4:**
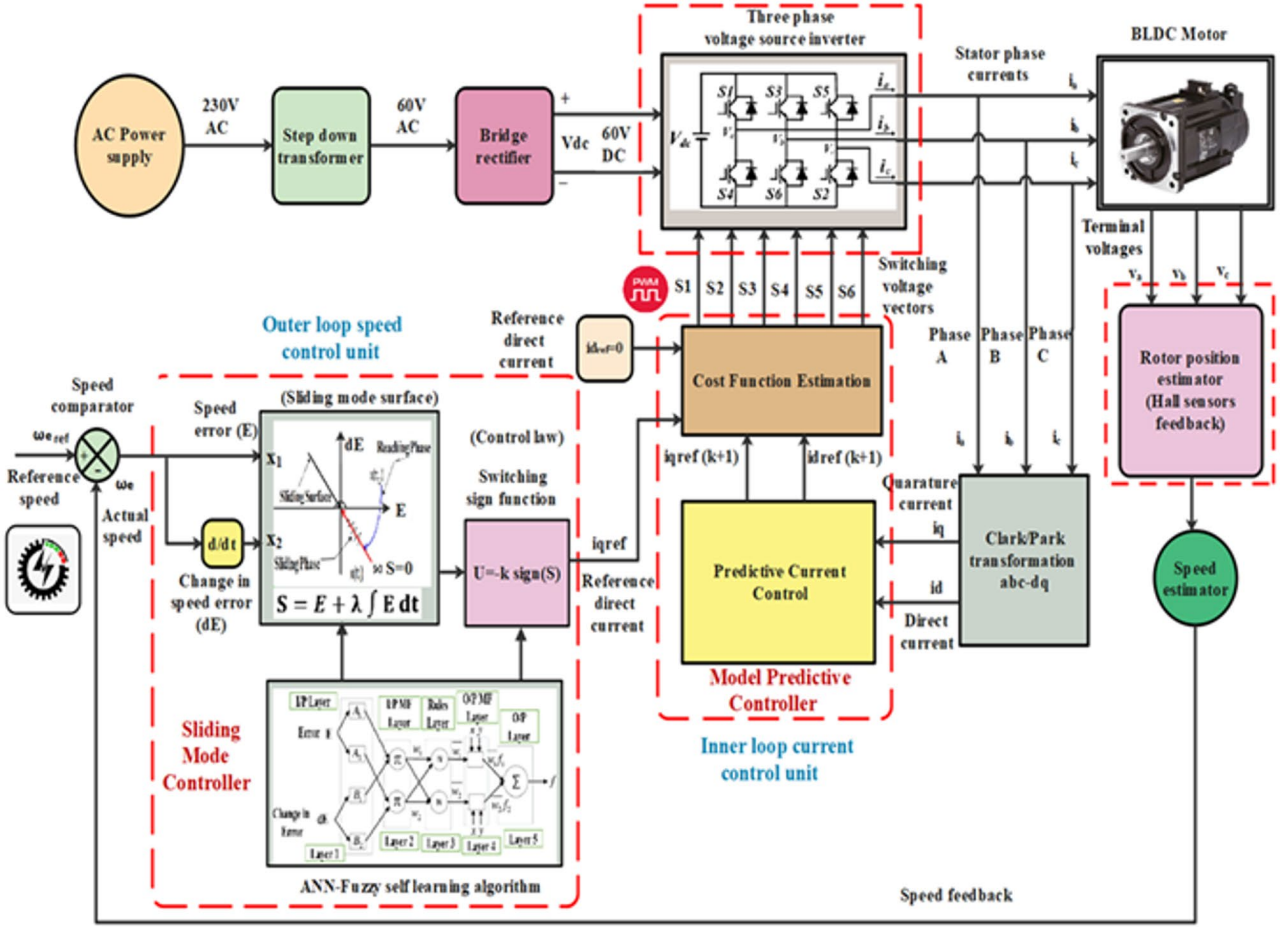
Proposed sliding mode—model predictive controller structure of BLDC motor in EVs.

This study presents a multiloop SMC–MPC controller for enhancing BLDC motor dynamic behavior in EV applications, developed on a hybrid SMC–MPC–ANN–FLC framework recently filed as an Indian patent (Application No. 202541082634). As the outer loop, the sliding mode controller monitors the BLDC motor’s velocity and compensates for load variations to ensure accurate alignment of the motor’s velocity with the reference setpoint. Meanwhile, the MPC controller functions as the inner loop, regulating the current to reduce the torque ripple in the BLDC motor. The design leverages the SM controller’s rapid response and resilience to uncertainty, as well as the MP controller’s ability to define system constraints. Integrating these controllers creates a powerful and effective control strategy for BLDC motors, ensuring precise speed regulation, robust performance, and reduced torque oscillations. This strategy effectively manages the demanding conditions of electric motors in EVs. Figure 4 illustrates the control scheme for the proposed SMC-MPC strategy. Initially, the rectifier transforms the AC supply into DC, which is then fed to the IGBT-based VSI via a 60 V DC link to power the motor. The VSI commutation angles facilitate smooth rotor rotation, while three hall sensors provide rotor position and speed feedback for motor control. The VSI gate signals are considered control variables, while the BLDC motor speed is regarded as the controlled variable. The three-phase VSI subsequently delivers the optimized potential to the BLDC motor according to the commands from the multiloop SMC-MPC controller. Examine the phase currents i_a_, i_b_, and i_c_, evaluate the rotor speed (N_r_) and mechanical angle (θ_m_), and derive the angular speed (ω_e_) and electrical degree (θ_e_). Subsequently, Clarke and Park’s transformations are utilized to calculate the dq-axis currents, i_d_ and i_q_, with the d-axis current reference point, i_dref_,​ maintained at zero. By contrasting the reference speed with the motor’s angular velocity ω_e_​, the q-axis current reference point I_qref_​ is determined, and the resultant control error is processed through the speed outer-loop SM controller. Besides, the current regulation is determined using an inner loop MP controller, which analyzes the prediction model through a cost function.

Based on the sequence of control input predicted currents (i_qref_) and (i_dref_) at the k + 1 instant, a cost function is designed to calculate the minimum cost and select the optimal voltage vectors (S_1_ to S_6_). By forecasting the motor system’s future behavior in the upcoming sampling period using these potential voltage vectors, the model predictive controller significantly enhances stator current waveforms and effectively mitigates torque fluctuations. The controller operates in a closed-loop system, continually optimizing predictions and control actions based on real-time motor current, voltage, and rotor position data.

### Outer loop sliding mode speed controller design

The sliding mode controller design is analyzed through four essential components: state equation formulation, determination of the sliding surface, selection of the control law with Lyapunov-based and reference current generation. This methodology ensures robust and accurate speed regulation of BLDC motors under varying dynamic conditions. Central to the SMC design is the formulation of a sliding surface, which governs the desired error dynamics and directs system trajectories toward the reference point.

#### State variable equation formulation

During BLDC motor operation, the Hall-effect sensor estimates the rotor position, triggering the commutation logic and coordinating the PWM inverter accordingly. Equations (16) and (17) delineate the speed error (E) and its change (dE), representing the deviation from the desired speed. In this BLDC motor system, E and dE are chosen as state variables, $\:{\mathrm{x}}_{1}$ and $\:{\mathrm{x}}_{2}$.

16 $$\:\mathrm{E}={\mathrm{x}}_{1}={{\upomega\:}}_{\mathrm{e}\:\mathrm{r}\mathrm{e}\mathrm{f}}\:-\:{{\upomega\:}}_{\mathrm{e}}\:$$

17 $$\:{\mathrm{d}\mathrm{E}=\mathrm{x}}_{2}=\dot{\mathrm{X}1}=-\dot{{{\upomega\:}}_{\mathrm{e}}}$$

where $\:{{\upomega\:}}_{\mathrm{e}\:\mathrm{r}\mathrm{e}\mathrm{f}}\:$is the desired speed, $\:{{\upomega\:}}_{\mathrm{e}}$ is the actual speed ($\:{{\upomega\:}}_{\mathrm{e}}\:$= P $\:{{\upomega\:}}_{\mathrm{m}\:}$), and E & dE represent the speed error and its change in the BLDC motor. According to Eqs. (14) and (15), differentiating $\:{\mathrm{x}}_{1}$ and $\:{\mathrm{x}}_{2}$ in Eqs. (16) and (17) produce,

$$\:\dot{\mathrm{X}1}=-\dot{{{\upomega\:}}_{\mathrm{e}}}=\frac{\mathrm{P}}{\mathrm{J}\mathrm{m}}\left[-1.5\mathrm{P}{{\uppsi\:}}_{\mathrm{r}}{\:\mathrm{i}}_{\mathrm{q}}+{\mathrm{T}}_{\mathrm{L}}+\:{{\mathrm{B}}_{\mathrm{m}}{\upomega\:}}_{\mathrm{m}}\right]$$

18 $$\:\dot{\mathrm{X}1}=-\dot{{{\upomega\:}}_{\mathrm{e}}}=\frac{\mathrm{P}}{\mathrm{J}\mathrm{m}}[-1.5\mathrm{P}{{\uppsi\:}}_{\mathrm{r}}{\:\mathrm{i}}_{\mathrm{q}}+{\mathrm{T}}_{\mathrm{L}}+{\mathrm{B}}_{\mathrm{m}}\:\frac{{{\upomega\:}}_{\mathrm{e}}}{\mathrm{P}}]$$

$$\:\dot{\mathrm{X}2}={-\ddot{{{\upomega\:}}_{\mathrm{e}}}=-\frac{1.5\:{\mathrm{P}}^{2}{{\uppsi\:}}_{\mathrm{r}\:{\:\mathrm{i}}_{\mathrm{q}}}}{\mathrm{J}\mathrm{m}}+\frac{{\mathrm{B}}_{\mathrm{m}\:\dot{{{\upomega\:}}_{\mathrm{e}}}}}{{\mathrm{J}}_{\mathrm{m}}}\:\:\:\:\:}_{\:}$$

19 $$\:\dot{\mathrm{X}2}={-\ddot{{{\upomega\:}}_{\mathrm{e}}}=-\frac{1.5\:{\mathrm{P}}^{2}{{\uppsi\:}}_{\mathrm{r}\:\dot{{\:\mathrm{i}}_{\mathrm{q}}}\:}}{\mathrm{J}\mathrm{m}}-\:\frac{{\mathrm{B}}_{\mathrm{m}\:}}{{\mathrm{J}}_{\mathrm{m}}}\:{\mathrm{x}}_{2}\:\:\:\:\:\:}_{\:}$$

where $\:{\mathrm{x}}_{1}$and $\:{\mathrm{x}}_{2\:}$are the state variables, and $\:\dot{X1}$ and $\:\dot{X2}\:$are the state equations of the BLDC motor control systems. These equations describe the speed control dynamics of the BLDC motor, under the assumption of constant system parameters (J_m_, B_m_ and ψ_r_) and bounded disturbances such as load torque T_L_, which are reasonable for initial control law formulation.

#### Sliding surface determination

The first step in the SMC design is the formulation of a sliding surface ‘S’, which defines the desired error dynamics. For current control in BLDC motors, a common sliding surface^7,29^ is given by,

$$\:\mathrm{S}={{\upomega\:}}_{\mathrm{e}\:\mathrm{r}\mathrm{e}\mathrm{f}}\:-\:{{\upomega\:}}_{\mathrm{e}}$$

The sliding surface is formulated to encapsulate the tracking error dynamics in the speed control loop and is typically expressed as,

20 $$\:S=E+\lambda\:\:\int\:E\:dt={(\omega\:}_{e\:ref}\:-{\omega\:}_{e})\:+\lambda\:\:\int\:{(\omega\:}_{e\:ref}-\:{\omega\:}_{e})dt\:$$

where ω_e_​ is the rotor speed, ω_e ref_​ is the reference speed, E is the speed error, and λ is a positive design parameter that governs the convergence dynamics.

The second step involves differentiating Eq. (20) to obtain the derivative of the sliding surface, expressed as,

$$\:\dot{\mathrm{S}}=\:\dot{\mathrm{E}}+\:{\uplambda\:}\mathrm{E}=\:\dot{{({\upomega\:}}_{\mathrm{e}\:\mathrm{r}\mathrm{e}\mathrm{f}}}-\:\dot{{{\upomega\:}}_{\mathrm{e}})}+{\uplambda\:}\:{({\upomega\:}}_{\mathrm{e}\:\mathrm{r}\mathrm{e}\mathrm{f}}\:-\:{{\upomega\:}}_{\mathrm{e}})$$

Assume $\:\dot{{\:{\upomega\:}}_{\mathrm{e}\:\mathrm{r}\mathrm{e}\mathrm{f}}}=0\:$(constant reference speed)

21 $$\:\dot{\mathrm{S}}=\dot{{{\upomega\:}}_{\mathrm{e}}}+\:{\uplambda\:}\mathrm{s}\:\:\:$$

To ensure sliding mode, the reaching condition $\:\dot{\mathrm{S}\mathrm{S}}<0\:$must be satisfied. This condition ensures the system trajectory converges onto the sliding manifold S = 0.

#### Establishing control and law: Lyapunov-based stability analysis

To ensure that the system’s state transitions toward and remains on the sliding surface, a suitable control law must be selected to satisfy the sliding condition. Accordingly, the following control law is proposed,

$$\:\dot{\mathrm{S}}=-\mathrm{k}\:\mathrm{s}\mathrm{i}\mathrm{g}\mathrm{n}\left(\mathrm{S}\right)$$

22 $$\:\mathrm{S}\dot{\mathrm{S}}=\mathrm{s}\left(-\mathrm{k}\:\mathrm{s}\mathrm{i}\mathrm{g}\mathrm{n}\left(\mathrm{S}\right)\right)<0$$

For smoother convergence, $\:\dot{\mathrm{S}}=-\mathrm{k}\:\mathrm{s}\mathrm{i}\mathrm{g}\mathrm{n}\left(\mathrm{S}\right)-\:{\uplambda\:}\mathrm{s}$

23 $$\:\dot{\mathrm{S}\mathrm{S}}=\mathrm{S}(-\mathrm{k}\:\mathrm{s}\mathrm{i}\mathrm{g}\mathrm{n}\left(\mathrm{S}\right)-\:{\uplambda\:}\mathrm{s}\:)<0\:$$

This guarantees the system trajectory will reach and remain on the sliding surface, thus ensuring asymptotic stability^7,30^.

To establish system stability rigorously, we establish a Lyapunov prospective function:

24 $$\:\mathrm{V}\left(\mathrm{s}\right)=\frac{1}{2}\:{\mathrm{s}}^{2}$$

V(S) taking the derivative with respect to time,

25 $$\:\dot{\:\mathrm{V}}\left(\:\mathrm{S}\right)=\mathrm{S}\dot{\mathrm{S}}=\mathrm{s}\left(-\mathrm{k}\:\mathrm{s}\mathrm{i}\mathrm{g}\mathrm{n}\left(\mathrm{S}\right)-\:{\uplambda\:}\mathrm{S}\right)=\:-\mathrm{k}\:\mid\:\mathrm{S}\mid\:\:{\uplambda\:}\:{\mathrm{s}}^{2}<0$$

The negative definiteness of $\:\dot{\:\mathrm{V}}\left(\:\mathrm{S}\right)$affirms the global asymptotic stability of the closed-loop system with the proposed control law, even when faced with bounded disturbances like load torque variations. Therefore, the designed SMC ensures that the speed tracking error approaches zero.

#### Generation of reference current

The BLDC motor’s mechanical dynamics are governed by,

26 $$\:\mathrm{J}\mathrm{m}\:\dot{{{\upomega\:}}_{\mathrm{e}}}+\mathrm{B}\mathrm{m}\:{\upomega\:}\mathrm{m}=\:(\mathrm{T}\mathrm{e}-\mathrm{T}\mathrm{L}\:)$$

Here, $\:\mathrm{T}\mathrm{e}=1.5\:\mathrm{P}{{\uppsi\:}}_{\mathrm{r}\:\:}{\mathrm{i}}_{\mathrm{q}\:\:}$Solving for i_q_, we get,

27 $$\:{\mathrm{i}}_{\mathrm{q}\:\:}=\frac{0.666\:(\mathrm{J}\mathrm{m}\:\dot{{{\upomega\:}}_{\mathrm{e}}}+\:\mathrm{B}\mathrm{m}\:{\upomega\:}\mathrm{m}+\:\mathrm{T}\mathrm{L}\:)}{\mathrm{P}{{\uppsi\:}}_{\mathrm{r}}}$$

To synthesize the control law within the SMC framework, the load torque is treated as a bounded disturbance. The reference-axis current is constructed based on the sliding surface condition in Eq. (22). Substituting the expression from Eq. (21) and enforcing Eq. (22) yields the following reference current,

28 $$\:{\mathrm{i}}_{\mathrm{q}\:\mathrm{r}\mathrm{e}\mathrm{f}}=\frac{0.666\:(\mathrm{J}\mathrm{m}\left(\:{\uplambda\:}\:\mathrm{S}+\:\:\mathrm{k}\:\mathrm{s}\mathrm{i}\mathrm{g}\mathrm{n}\left(\mathrm{S}\right)\right)+\:\mathrm{B}\mathrm{m}{\upomega\:}\mathrm{m}+\:\mathrm{T}\mathrm{L})\:}{\mathrm{P}{{\uppsi\:}}_{\mathrm{r}}}$$

where k > 0 and $\:{\uplambda\:}\:$> 0 are design parameters. The expression in Eq. (28) encapsulates the torque dynamics necessary to enforce the sliding condition $\:\dot{\mathrm{S}}=-\mathrm{k}\:\mathrm{s}\mathrm{i}\mathrm{g}\mathrm{n}\left(\mathrm{S}\right)-\:{\uplambda\:}\mathrm{s}$, ensuring the system trajectory converges robustly to the sliding manifold and maintaining tracking performance under external perturbations. The load torque is considered as a bounded disturbance, and the SMC ensures robustness without requiring online estimation of T_L_.

### Inner loop model predictive current controller design

The design of the inner-loop Model Predictive Current Controller is critical for ensuring high-fidelity current tracking and torque control in BLDC motor drives. The MPC strategy presented in this work is systematically structured into two key stages: (i) the one-step-ahead prediction of the stator current response, (ii) the formulation and real-time evaluation of a cost function to determine optimal switching actions (iii) MPC formulation with convergence capabilities, and (iv) Direct discrete-time control and synchronization with the outer loop. By using a Finite Control Set MPC (FCS-MPC) technique, the suggested controller ensures a hardware-friendly and computationally efficient implementation by generating inverter switching signals directly without the need for traditional PWM modulation.

#### Phase current prediction

The BLDC motor’s continuous-time dynamics are first stated in the stationary α–β reference frame to provide a theoretical basis for the Model Predictive Current Controller (MPC). The stator voltage equations are as follows under the conventional modelling assumptions of linearity, nominal motor specifications, and constant load torque:

29 $$\:{\mathrm{V}}_{{\upalpha\:}}\:=\:{\mathrm{R}}_{\mathrm{s}}\:{\mathrm{i}}_{{\upalpha\:}}+\mathrm{L}\frac{\mathrm{d}}{\mathrm{d}\mathrm{t}}{\mathrm{i}}_{{\upalpha\:}}+{\mathrm{e}}_{{\upalpha\:}}\:$$

30 $$\:{\mathrm{V}}_{{\upbeta\:}}\:=\:{\mathrm{R}}_{\mathrm{s}}\:{\mathrm{i}}_{{\upbeta\:}}+\mathrm{L}\frac{\mathrm{d}}{\mathrm{d}\mathrm{t}}{\mathrm{i}}_{{\upbeta\:}}+{\mathrm{e}}_{{\upbeta\:}}\:$$

where the stator voltage, current, and back-EMF components are represented by V_β_, i_β,_ and e_β_.

 and L represent stator resistance and inductance.

The control input computed at the current sample instant is applied effectively in the next time step in real-time embedded systems because of inherent actuation and computation constraints. Control accuracy and stability are impacted by this one-step delay, which becomes more noticeable at lower sampling rates. To address this, a discrete-time prediction model is derived by discretizing the continuous-time electrical dynamics of the BLDC motor using the first-order Euler method^31^.

31 $$\:{\mathrm{i}}_{{\upalpha\:}\:(\mathrm{k}+1)}={\mathrm{i}}_{{\upalpha\:}\:}\left(\mathrm{k}\right)+\frac{{\mathrm{T}}_{\mathrm{s}}}{\mathrm{L}}\:(-\mathrm{R}{\mathrm{i}}_{{\upalpha\:}\:}\left(\mathrm{k}\right)+\:{\mathrm{V}}_{{\upalpha\:}}\left(\mathrm{k}\right)-{\mathrm{e}}_{{\upalpha\:}}\left(\mathrm{k}\right))\:$$

32 $$\:{\mathrm{i}}_{{\upbeta\:}\:(\mathrm{k}+1)}={\mathrm{i}}_{{\upbeta\:}\:}\left(\mathrm{k}\right)+\frac{{\mathrm{T}}_{\mathrm{s}}}{\mathrm{L}}\:(-\mathrm{R}{\mathrm{i}}_{{\upbeta\:}\:}\left(\mathrm{k}\right)+\:{\mathrm{V}}_{{\upbeta\:}}\left(\mathrm{k}\right)-{\mathrm{e}}_{{\upbeta\:}}\left(\mathrm{k}\right))\:$$

The cost function used to minimise tracking error is based on the one-step-ahead predictions i_α,β_(k + 1). The simulation includes dynamic load, back-EMF variation, and system disturbances, even though its derivation is predicated on perfect circumstances. Through online control weight adjustments, adaptive ANN and FLC modules improve robustness. Specifically, the one-step prediction for the dq-axis currents is as follows, Eqs. (33) and (34),

33 $$\:{\mathrm{i}}_{\mathrm{d}\:(\mathrm{k}+1)\left(\mathrm{p}\mathrm{r}\mathrm{e}\mathrm{d}\mathrm{i}\mathrm{c}\mathrm{t}\mathrm{i}\mathrm{o}\mathrm{n}\right)}=\:\left(1-\frac{{\mathrm{R}}_{\mathrm{s}}}{\mathrm{L}\mathrm{s}\mathrm{d}}{\mathrm{T}}_{\mathrm{s}}\:\right){\mathrm{i}}_{\mathrm{d}\:}\left(\mathrm{k}\right)+\frac{\mathrm{L}\mathrm{s}\mathrm{q}}{\mathrm{L}\mathrm{s}\mathrm{d}}{\mathrm{T}}_{\mathrm{s}}\:{{\upomega\:}}_{\mathrm{e}}\:{\mathrm{i}}_{\mathrm{q}\:}\left(\mathrm{k}\right)+\frac{{\mathrm{T}}_{\mathrm{s}}}{\mathrm{L}\mathrm{s}\mathrm{d}}\:{\mathrm{V}}_{\mathrm{s}\mathrm{d}}\left(\mathrm{k}\right)$$

34 $$\:{\mathrm{i}}_{\mathrm{q}\:(\mathrm{k}+1)\left(\mathrm{p}\mathrm{r}\mathrm{e}\mathrm{d}\mathrm{i}\mathrm{c}\mathrm{t}\mathrm{i}\mathrm{o}\mathrm{n}\right)}=\:\left(1-\frac{{\mathrm{R}}_{\mathrm{s}}}{\mathrm{L}\mathrm{s}\mathrm{q}}{\mathrm{T}}_{\mathrm{s}}\:\right){\mathrm{i}}_{\mathrm{q}}\left(\mathrm{k}\right)-\frac{\mathrm{L}\mathrm{s}\mathrm{d}}{\mathrm{L}\mathrm{s}\mathrm{q}}{\mathrm{T}}_{\mathrm{s}}\:{{\upomega\:}}_{\mathrm{e}}\:{\mathrm{i}}_{\mathrm{d}\:}\left(\mathrm{k}\right)+\frac{{\mathrm{T}}_{\mathrm{s}}}{\mathrm{L}\mathrm{s}\mathrm{q}}\:{\mathrm{V}}_{\mathrm{s}\mathrm{q}}\left(\mathrm{k}\right)-{\mathrm{T}}_{\mathrm{s}}{{\upomega\:}}_{\mathrm{e}}\:\frac{{{\uppsi\:}}_{\mathrm{r}}}{\mathrm{L}\mathrm{s}\mathrm{q}}$$

where$\:\:{\mathrm{i}}_{\mathrm{d}\:\left(\mathrm{k}+1\right)\:\left(\mathrm{p}\mathrm{r}\mathrm{e}\mathrm{d}\mathrm{i}\mathrm{c}\mathrm{t}\mathrm{i}\mathrm{o}\mathrm{n}\right)}\:\mathrm{a}\mathrm{n}\mathrm{d}\:{\mathrm{i}}_{\mathrm{q}\:\left(\mathrm{k}+1\right)\:\left(\mathrm{p}\mathrm{r}\mathrm{e}\mathrm{d}\mathrm{i}\mathrm{c}\mathrm{t}\mathrm{i}\mathrm{o}\mathrm{n}\right)}\:$represent the estimated current values at the (k + 1) time step along the d-axis and q-axis, respectively, $\:\:{\mathrm{i}}_{\mathrm{d}\:\left(\mathrm{k}\right)\:\:}\mathrm{a}\mathrm{n}\mathrm{d}\:{\mathrm{i}}_{\mathrm{q}\:\left(\mathrm{k}\right)}\:$represent the measured current values in the d-axis and q-axis, respectively, whereas $\:{\mathrm{T}}_{\mathrm{s}}\:$is the sampling interval.

In a two-level voltage source inverter, there exist six feasible active switching states (S_1_​ through S_6_​). The corresponding six voltage vectors are substituted into Eqs. (33) and (34) to predict the resulting stator current behavior at time step k + 1. This prediction process forms the basis of the FCS-MPC framework, wherein all candidate switching vectors are evaluated using a cost function, and the optimal vector is selected for implementation. The predictive model is updated at every sampling instance, enabling dynamic adaptation of control actions in real time. Notably, the voltage vectors correspond to actual inverter states, thereby eliminating the need for modulation stages and facilitating direct gate signal generation.

#### Cost function formulation

The selection of the optimal switching state is governed by a cost function that quantifies the deviation between predicted and reference currents. For torque control in BLDC motors, the d-axis current is set to zero to ensure efficient field orientation, while the q-axis current directly contributes to torque production and is regulated based on the speed error^32^. The cost function ($\:g$) evaluates control actions based on stator current errors, as expressed in Eq. (35).

35 $$\:\mathrm{g}={[\:{\mathrm{i}}_{\mathrm{d}\:\mathrm{r}\mathrm{e}\mathrm{f}}-\:{\mathrm{i}}_{\mathrm{d}\:\left(\mathrm{k}+1\right)\:\left(\mathrm{p}\mathrm{r}\mathrm{e}\mathrm{d}\mathrm{i}\mathrm{c}\mathrm{t}\mathrm{i}\mathrm{o}\mathrm{n}\right)}]}^{2}+{\left[\:{\mathrm{i}}_{\mathrm{q}\:\mathrm{r}\mathrm{e}\mathrm{f}}-\:{\mathrm{i}}_{\mathrm{q}\:\left(\mathrm{k}+1\right)\:\left(\mathrm{p}\mathrm{r}\mathrm{e}\mathrm{d}\mathrm{i}\mathrm{c}\mathrm{t}\mathrm{i}\mathrm{o}\mathrm{n}\right)}\right]}^{2}\:$$

where$\:\:{\mathrm{i}}_{\mathrm{d}\:\left(\mathrm{r}\mathrm{e}\mathrm{f}\right)\:\:}\mathrm{a}\mathrm{n}\mathrm{d}\:{\mathrm{i}}_{\mathrm{q}\:\left(\mathrm{r}\mathrm{e}\mathrm{f}\right)}\:\:$represent the reference current values of the d-axis and q-axis, respectively.

**Fig. 5:**
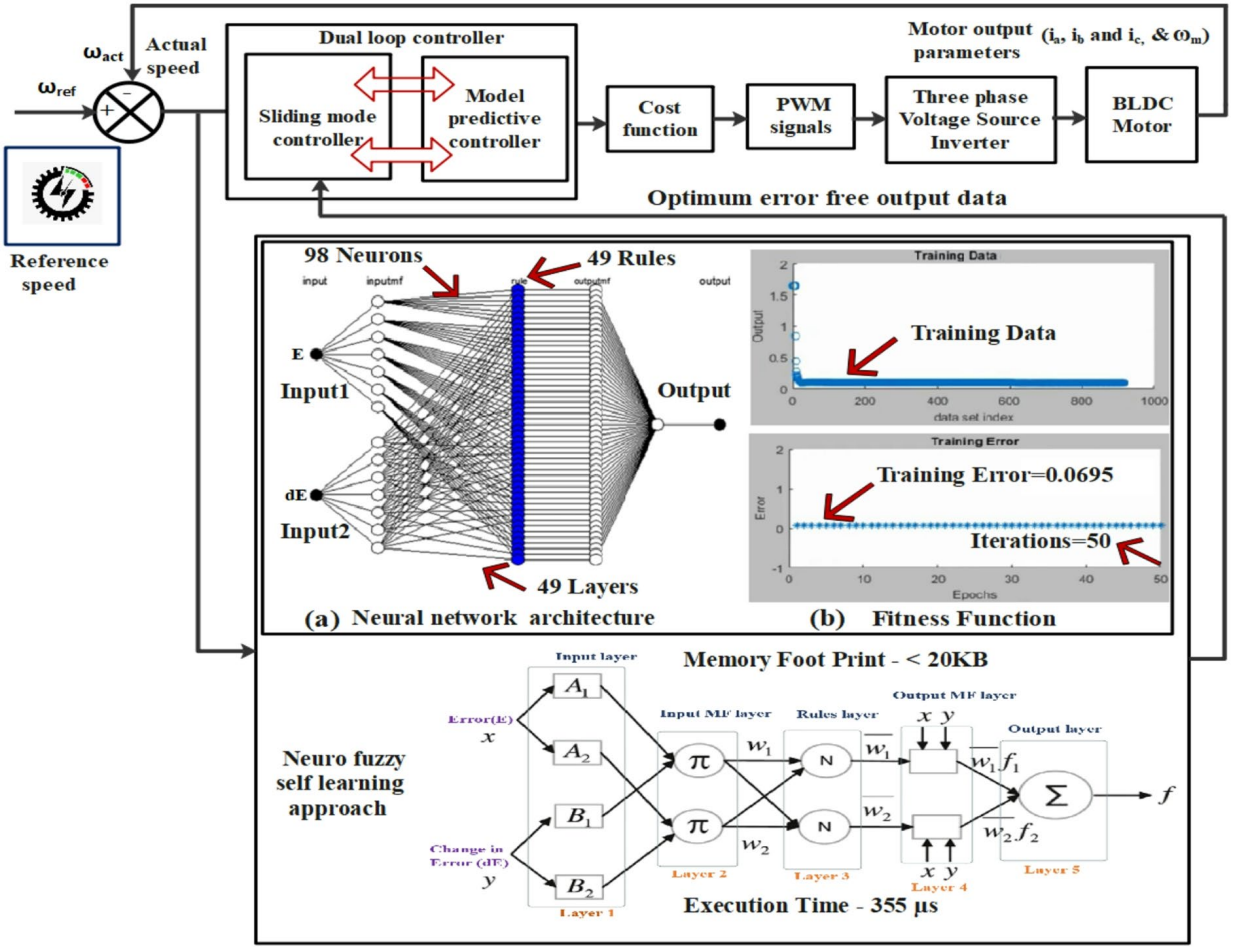
An ANN-Fuzzy self-learning SM- MP controller architecture (**a**) Neural network architecture (**b**) Fitness function.

At each sampling interval, the controller evaluates this cost function for all six predicted current pairs derived from the inverter’s switching states. The voltage vector that yields the minimum cost is identified as the optimal control input. Crucially, this voltage vector corresponds to a specific set of high/low logic states across the inverter legs. So, the chosen switching pattern is directly applied to the three-phase inverter gate drivers, which means that pulse-width modulation does not exist and the system is compatible with digital logic controllers, as shown in Fig. 5.

This control framework establishes a rigorous and analytically grounded correspondence between cost function minimization and the discrete control action executed by the inverter. The MPC algorithm not only minimizes the current tracking error but also selects the optimal switching vector that directly determines the instantaneous state of the power converter. The discrete control action improves the responsiveness and efficiency of the system by removing the computational overhead usually associated with traditional modulation approaches and enabling real-time application within embedded systems.

Importantly, the inner-loop MPC controller operates in coordination with the outer-loop Sliding Mode Speed Controller (SMC), which dynamically adjusts the reference q-axis current (i_qref_​) based on the motor’s speed error. The SMC ensures robust speed regulation under dynamic conditions and disturbances, while the inner-loop MPC guarantees precise tracking of the stator current reference generated by the outer loop. The system’s torque control precision and disturbance rejection capabilities are improved by this structured dual-loop structure, which makes it especially appropriate for electric vehicle drive applications that demand high-performance, real-time operation.

#### MPC formulation convergence capabilities

The convergence of the FCS-MPC algorithm is governed by the properties of the cost function and the predictive model. Since the cost function g in (35) is strictly convex over the finite set of discrete switching vectors, and all six candidate vectors are exhaustively evaluated at each sampling step, a global minimum is guaranteed to be found within the finite horizon of prediction. Furthermore, the one-step prediction model ensures boundedness and continuity in the predicted current trajectories, under the assumption of bounded system parameters and inverter constraints. As a result, the selected control action at each time step always reduces the norm of the tracking error vector, leading to step-by-step convergence toward the reference current values.

Although FCS-MPC does not solve an infinite-horizon optimum control problem directly, its greedy minimisation technique guarantees that the tracking error stays confined and asymptotically approaches zero, provided that the sampling time T_s_​ is sufficiently small, the stator inductance and resistance are correctly identified, the input-output latency is either compensated or very little, and the system is observable. This guarantees recursive feasibility, where each control action remains valid over subsequent time steps, enabling robust current tracking even in the presence of system perturbations or load disturbances.

#### Direct discrete control and synchronisation with the outer loop

The MPC framework establishes a direct mapping between inverter switching states and optimal control actions. Since each voltage vector is tied to a specific combination of gate signals, no modulation stage is required. The selected control action is directly fed to the gate drivers of the three-phase voltage source inverter referred to in Fig. 4, thus reducing computational overhead and improving response time.

Furthermore, the inner-loop MPC works in concert with the outer-loop SMC, which continuously modifies IQ_ref_ in response to the dynamics of speed errors in real time. Both torque response precision and resilience under various operating situations are improved by this dual-loop coordination. While the SMC guarantees asymptotic speed regulation, the MPC ensures instantaneous current enforcement, making the overall control architecture particularly suitable for high-performance electric vehicle drive applications.

### Adaptive hybrid SMC-MPC control using ANN-FLC integration

Conventional control methods exhibit inherent limitations in adaptability, tuning accuracy, and robustness when applied to BLDC motor drives operating under dynamic and uncertain conditions, such as varying loads and nonlinearities. To address these challenges, this study introduces a self-learning hybrid control architecture that integrates Sliding Mode Control (SMC), Model Predictive Control (MPC), Artificial Neural Networks (ANN), and Fuzzy Logic Control (FLC) as illustrated in Fig. 5a,b. This composite controller is specifically designed to improve speed regulation and torque pulsation mitigation in BLDC motor systems for electric vehicle (EV) applications.

While SMC is well recognized for its robustness and insensitivity to disturbances, it remains sensitive to the choice of its design parameters. Classical SMC suffers from chattering and lacks self-tuning capabilities when subjected to nonlinear system variations. To overcome these drawbacks, the sliding surface within the SMC structure is augmented using ANN, which functions as an intelligent estimator of the ideal sliding trajectory. The estimated sliding surface, denoted as s(t), is derived from the instantaneous system state and desired trajectory and is then passed as input to a fuzzy logic system. The FLC processes the ANN-estimated sliding variable and system state to compute a nonlinear adaptive control signal u(t). In this configuration, the ANN and FLC components do not replace the SMC but act as adaptive extensions that enhance the original SMC structure by embedding learning and self-adjustment capabilities into its decision-making process.

The mathematical representation of the interaction between Artificial Neural Networks (ANN) and fuzzy logic is captured by the Eqs. (36) and (37),

36 $$s(t) = f_{\mathrm{ANN}}\big(x(t),\, x_d(t),\, \Theta_{\mathrm{ANN}}\big)$$

37 $$u(t) = f_{\mathrm{FLC}}\big(s(t),\, x(t),\, \Theta_{\mathrm{FLC}}\big)                                                                         $$

where s(t) stands as the ANN estimated sliding mode surface, x(t) is the current system state, x_d_(t) is the desired system state, ϴ_ANN_ is the neural network parameters, u(t) stands as FLC control action, and ϴ_FLC_ is FLC parameters.

Significantly, the outer-loop SMC controller incorporates this adaptive ANN-FLC module. Its function is to adjust for modelling uncertainties and disturbances to dynamically update the sliding surface and increase control precision. Thus, contrary to any assumption that the SMC is negated, this structure explicitly preserves the foundational SMC behavior, while enabling a self-tuning mechanism for improved real-time adaptability.

The output of the ANN-FLC-enhanced SMC controller is the reference q-axis current iq ref​, which is dynamically adjusted based on the speed tracking error. This reference is then relayed to the inner-loop MPC controller. As described, the inner-loop utilizes an FCS-MPC formulation to predict future current states and select optimal inverter switching states that minimize the error between predicted and reference currents, thereby reducing torque ripple and enhancing current regulation accuracy.

This hierarchical dual-loop structure ensures both robust outer-loop speed tracking and high-resolution inner-loop torque control, wherein the outer loop is governed by an SMC controller augmented with ANN-FLC intelligence to enable real-time self-tuning and adaptive sliding surface estimation under varying motor dynamics, while the inner loop employs a FCS-MPC strategy to minimize stator current errors and determine optimal switching states at each sampling instant.

The ANN itself is constructed with a five-layer architecture as shown in Fig. 5a. The first layer processes the input signals (tracking error E and its derivative dE). The second layer maps these inputs into fuzzy sets through appropriate membership functions.

Logical rule formation occurs in the third layer using fuzzy inference mechanisms (e.g., AND operations), followed by rule weight normalization in the fourth layer. Finally, the fifth layer generates the output control signal based on fuzzy rules^33,34^.To enhance learning efficiency, clustering algorithms are employed to refine the fuzzy membership functions. Iterative parameter optimisation is possible using a hybrid learning method that combines least squares error estimates with backpropagation. This training process is governed by a fitness function that aims to reduce overshoot and improve response time in transient conditions.

As depicted in Fig. 5b, convergence is achieved within 50 training iterations, reaching a steady-state error tolerance of 0.0695. The ANN-FLC-enhanced controller comprising 98 neurons and 49 layers demonstrates faster convergence and better regulation performance than traditional fixed-parameter methods. Sliding surface parameters can be adaptively tuned to produce smooth dynamic responses, low oscillations, and minimum torque distortion. Two phases make up the training process, which ensures continuous online learning: forward propagation for node output updates and backward propagation for weight refinement^35,36^.

The proposed hybrid control strategy employs an Artificial Neural Network–Fuzzy Logic Controller (ANN-FLC) framework, which integrates a deep feedforward neural network consisting of 49 layers and 98 neurons to effectively capture the nonlinear dynamics of a BLDC motor across varying load and speed conditions. A FLC with 49 expert-defined rules complements this artificial neural structure through linguistic variable mappings, improving decision interpretability and facilitating robust handling of system uncertainties. The artificial neural network was trained on a dataset comprising more than 10,000 samples, derived from a high-fidelity BLDC motor model under diverse operating load conditions (0%, 50%, 100%), such as steady-state, acceleration, deceleration, and torque transients. The Levenberg–Marquardt algorithm was utilized for training, resulting in a mean squared error (MSE) of 0.0695, which demonstrates effective learning and adequate generalization. To further support generalization and prevent overfitting, the dataset was partitioned into training (70%), validation (15%), and testing (15%) subsets. The robustness of the trained model was validated by testing it under previously unencountered disturbances and variations in load. The primary training occurred offline; however, real-time adaptability is achieved via an embedded fuzzy self-tuning mechanism that dynamically modifies rule strengths during operation according to immediate motor performance.

The ability of the proposed hybrid architecture was evaluated by measuring computation performance using SIL simulations. With a memory footprint of less than 20 KB and an average execution time of 355 µs, the ANN-FLC-enhanced SMC-MPC controller is suitable for real-time embedded systems in electric vehicle applications. The numbers show compatibility with typical automotive microcontrollers under a 1-ms control loop limitation. The ANN and FLC modules were individually assessed, consuming approximately 135 µs and less than 10 KB of memory, respectively, as tabulated in Table 4. The integration with SMC (outer loop) and MPC (inner loop) components was optimized to maintain timing efficiency. Although the ANN possesses considerable depth, the compact architecture of the controller and its rule-based efficiency facilitate real-time inference without excessive demand on processing resources.

**Table 4:** Computational load and architecture of the ANN-FLC-Enhanced SMC-MPC controller.

Parameters	Execution time (in µs)	Architecture/configuration	Observations
SMC (outer loop speed control)	˜ 40 µs	First-order switching surface accompanied by a boundary layer	Rapid computation through algebraic decision principles
MPC (inner loop current control)	˜ 180 µs	Prediction horizon is set to 5, utilising an online quadratic programming solver based on the active-set method	Controls multivariable constraints, the primary computational burden
ANN module	˜ 75 µs	49 layers with 98 fully connected neurons	Deep presently compact; trained using Levenberg–Marquardt (MSE = 0.0695)
FLC module	˜ 60 µs	2 inputs and 1output, 49 fuzzy rules	Real-time implementation with optimisation through lookup tables is feasible
ANN-FUZZY module	˜ 135 µs	Interaction loop between ANN and FLC	Incorporates both forward and backward pass evaluations throughout the learning process
Total control loop	˜ 355 µs		Functions effectively within a 1 ms control interval on standard DSPs and MCUs

Further optimisation strategies have been implemented to diminish the embedded computational burden, including fixed-point realisation, fuzzy rule-base reduction, and lookup-table approximations. When compared to fixed-parameter methods, the ANN-FLC-enhanced Sliding Mode Model Predictive Controller (SMC-MPC) performs better dynamically and exhibits less torque ripple. The ANN adjusts sliding surface parameters to ensure a smooth transient response, minimum chattering, and stable electromagnetic torque. Furthermore, the controller is integrated with the inverter logic through Hall-effect rotor position sensors, which determine the switching vector sequence for energizing the stator phases. As presented in Table 5, the six-step commutation scheme is defined over discrete 60° electrical intervals, corresponding to the Hall sensor outputs (H_1_, H_2_, H_3_). This switching pattern controls the activation of IGBT switches (S_1_–S_6_), which sequentially energize the stator windings (i_a_, i_b_, i_c_), thereby creating a revolving magnetic field that is synchronized with the rotor position. The hybrid controller’s integration with this switching logic enables exact commutation while also helping to reduce torque distortion during sector changes. The ANN-FLC enhanced SMC-MPC controller exhibits high adaptability, computational efficiency, and robustness, making it a compelling solution for next-generation EV motor drive systems.

**Table 5:** Computational load and architecture of the ANN-FLC-enhanced SMC-MPC controller.

Switching Sequences (position of the rotor) in deg	Position sensors (Hall signals)			Decoded signals			Active switches		Stator phase excitation sequence			
	H_1_	H_2_	H_3_	E_a_	E_b_	E_c_	1	2	I_a_	I_b_	I_c_	
0°–60°	1	0	0	1	0	− 1	SW1	SW6	+ve	Off	− ve	
60°–120°	1	1	0	0	1	− 1	SW3	SW6	Off	+ve	− ve	
120°–180°	0	1	0	− 1	1	0	SW3	SW2	− ve	+ve	Off	
180°–240°	0	1	1	− 1	0	1	SW5	SW2	− ve	Off	+ve	
240°–300°	0	0	1	0	− 1	1	SW5	SW4	Off	− ve	+ve	
300°–360°	1	0	1	1	− 1	0	SW1	SW4	+ve	− ve	Off	

## MIL simulation-based comparative analysis of the SMC-MPC controller method and conventional controllers

An exhaustive MATLAB/Simulink simulation model validates the proposed SMC-MPC technique for attenuating torque oscillations and enhancing overall motor dynamics, with parameters detailed in Table 6. The simulation evaluates critical performance metrics, including rise time, settled time, peak shoot, and error in steady state. Additionally, the motor operation is evaluated based on stator current, electromagnetic torque, speed fluctuations, and torque variations under various operating conditions. This approach aims to mitigate the challenges of excessive overshoot, extended settling times, and heightened torque variation encountered in conventional BLDC motor drive systems used in EVs while assessing their performance under various torque loading scenarios.

### Time response analysis

**Fig. 6:**
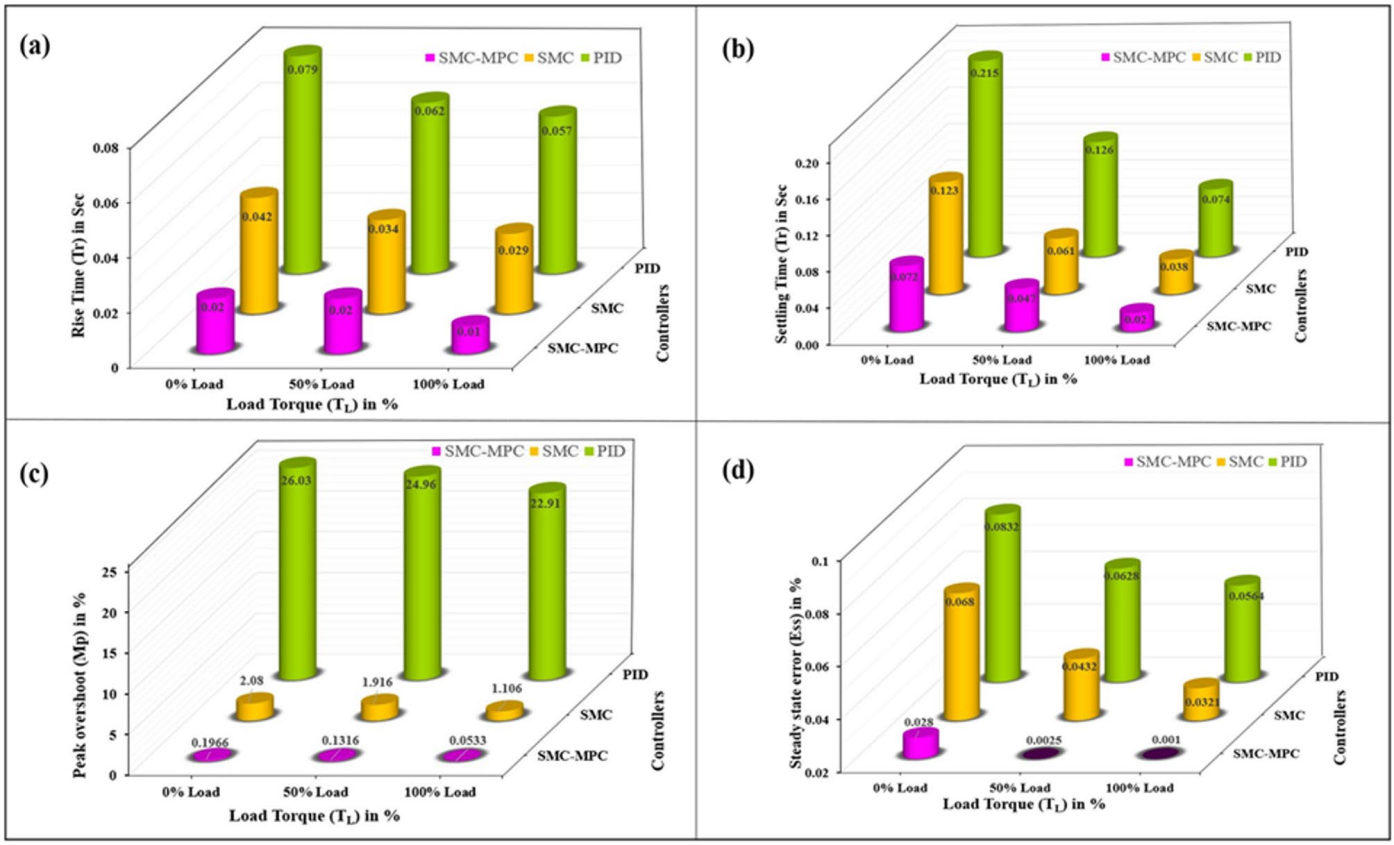
Comparison of time response analysis of various controllers at different load conditions (**a**) Rise time (**b**) Settling time (**c**) Peak overshoot (**d**) Steady state error.

Time-domain response assessment plays a pivotal role in mitigating torque pulsation within BLDC motors for EV drives, as it evaluates the system’s agility and stability in reacting to variations in load and control inputs, ensuring rapid and consistent motor performance. By examining metrics such as overshoot, rise time, and settling time, engineers can refine performance to enhance overall efficiency and stability. The proposed controller’s parameters are evaluated and compared with PID and SM controllers under different load conditions (0%, 50%, 100%) at 3000 rpm for (0–1 s), as shown in Fig. 6.

#### Rise time (t_r_)

**Fig. 7:**
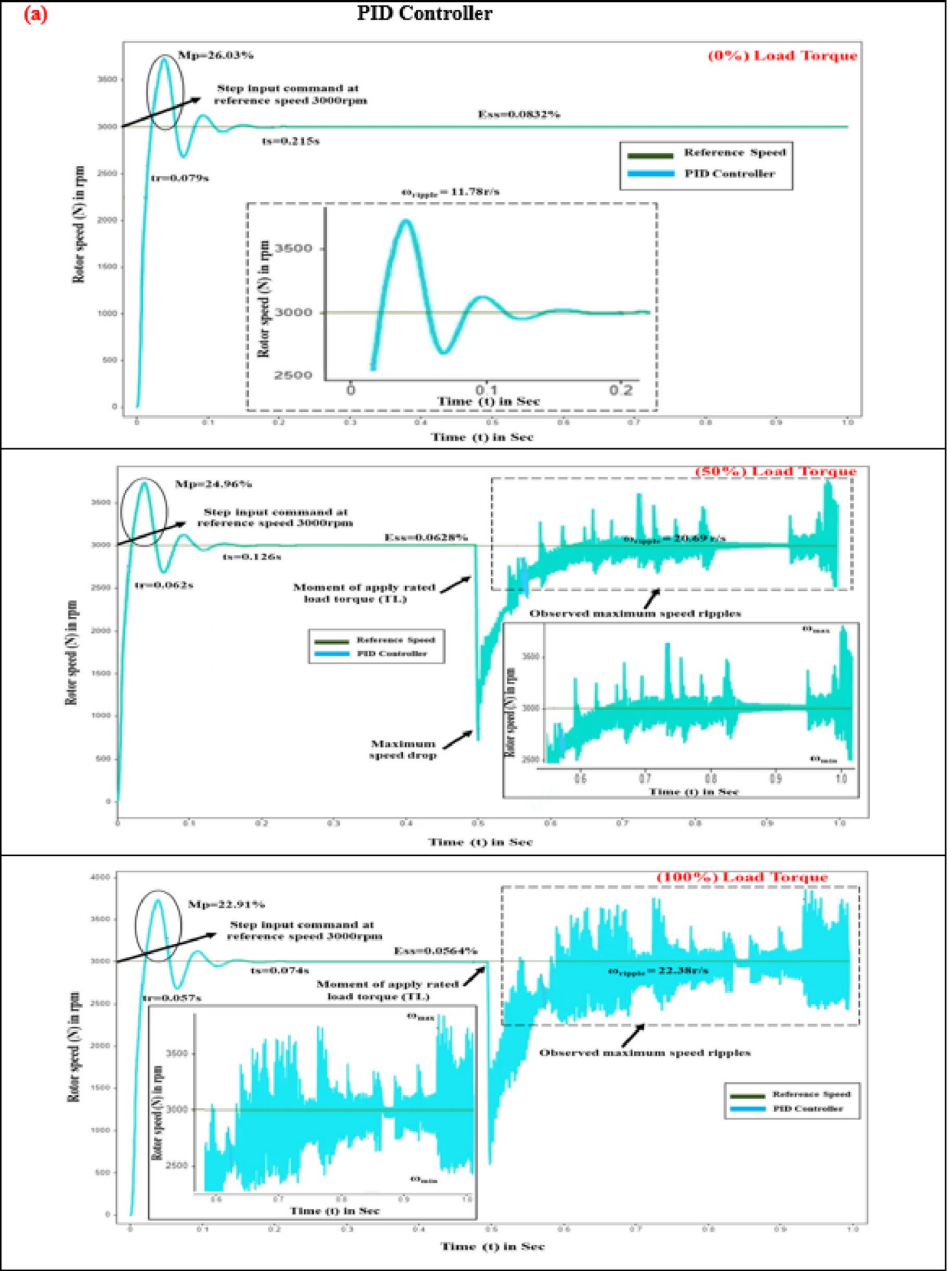

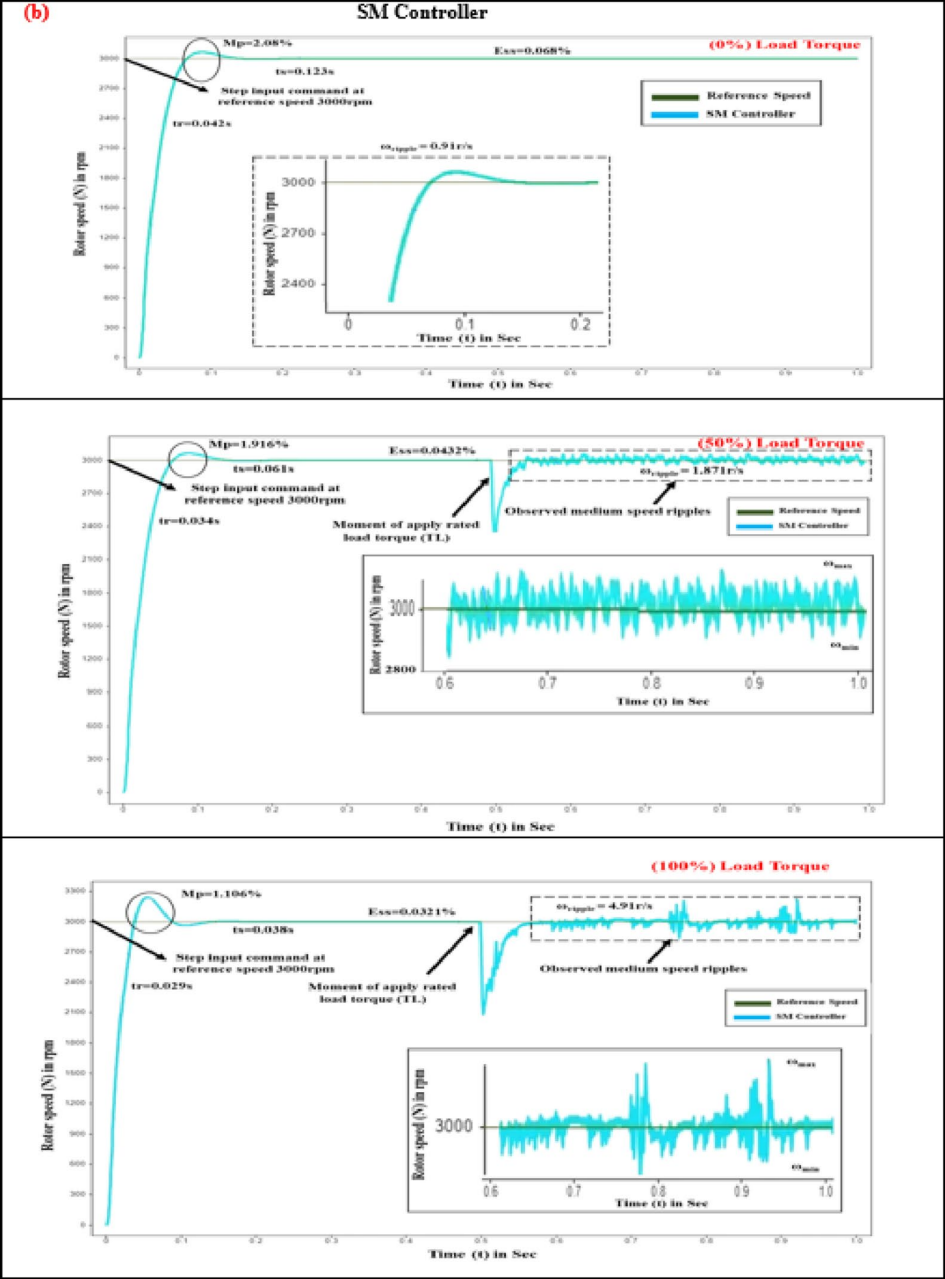

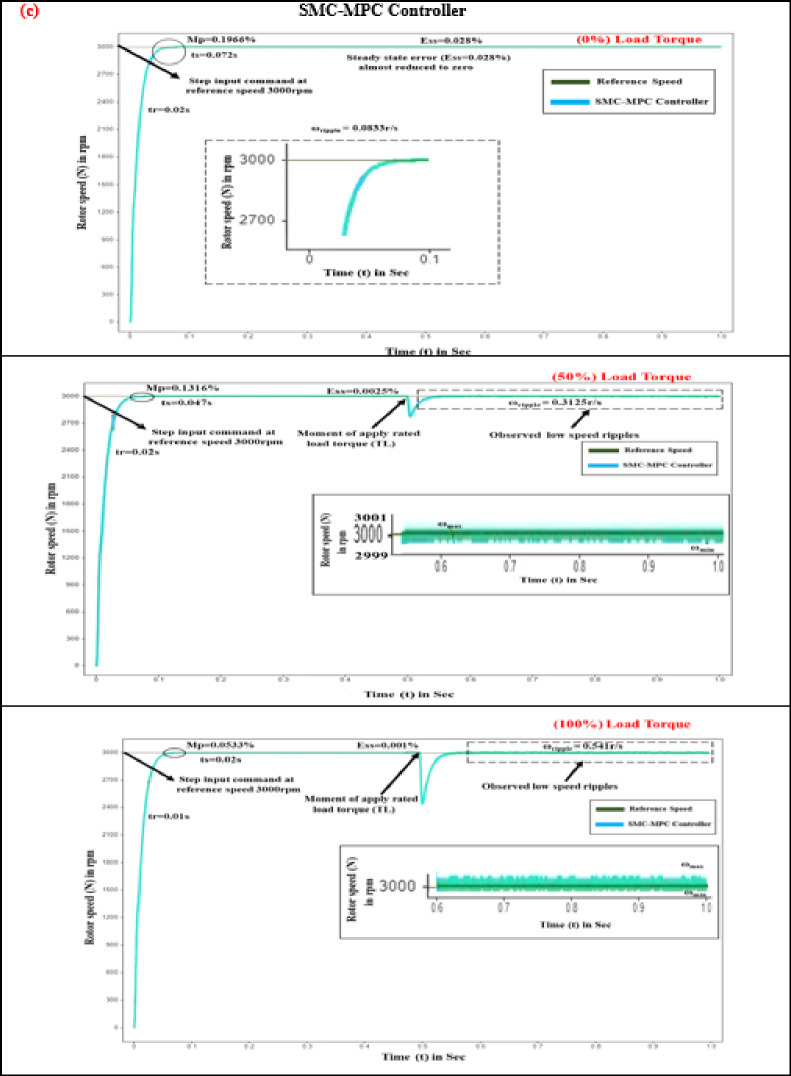
Rotor speed response of BLDC motor at different load conditions (**a**) PID controller. (**b**) SM controller. (**c**) SMC-MPC controller.

Evaluating how quickly a motor responds to control inputs is critical, with rise time serving as a key measure of this performance. This rapid response is vital for maintaining motor efficiency, reducing torque ripple, and ensuring overall system stability. Owing to the steep voltage change resulting from the variable frequency drive unit, the control voltage requires a finite period to transition between its minimum and maximum values. Nevertheless, advanced power conversion systems significantly shorten these periods of transition^37–39^. As illustrated in Fig. 7, at 3000 rpm and under zero load torque conditions, the BLDC motor demonstrated rise times of 0.079 s for the PID controller and 0.042 s for the SM controller.

**Table 6:** Comparative study of time response analysis in SMC-MPC and conventional controllers.

Load torque (T_L_) state in (%)	Time response metrics	Different controller designs		
		PID	SMC	SMC-MPC
No Load torque (0%)	Rise time (s)	0.079	0.042	0.02
	Settling time (s)	0.215	0.123	0.072
	Peak overshoot (%)	26.03	2.08	0.1966
	Steady-state error (%)	0.0832	0.068	0.028
Medium Load torque (50%)	Rise time (s)	0.062	0.034	0.02
	Settling time (s)	0.126	0.061	0.047
	Peak overshoot (%)	24.96	1.916	0.1316
	Steady-state error (%)	0.0628	0.0432	0.0025
Maximum Load Torque (100%)	Rise time (s)	0.057	0.029	0.01
	Settling time (s)	0.074	0.038	0.02
	Peak overshoot (%)	22.91	1.106	0.0533
	Steady-state error (%)	0.0564	0.0321	0.001

When subjected to 5 N-m and 10 N-m loads, the PID control system exhibited speed reductions of 752 rpm and 522 rpm, with recovery times of 0.042 s and 0.034 s, respectively. The SM controller, in contrast, exhibited more pronounced fluctuations. By implementing the SMC-MPC controller, speed drops were effectively reduced to 2810 rpm and 2480 rpm under load, with the quickest recovery times achieved at 0.02 s and 0.01 s, respectively. As shown in Table 6, the proposed controller demonstrated the fastest response and superior performance.

#### Settling time (t_s_)

In EV applications, the period required for the motor’s output torque to stabilize after a disturbance or command change is crucial, known as settling time, as smooth and stable torque delivery is essential for optimal vehicle performance and user experience^38–40^. During startup, the rotor speed climbs to 3000 rpm, and the torque adjusts to align with the load torque reference (0%).

Under zero-load conditions, the PID control system exhibits a settling period of 0.215 s, whereas the SM controllers achieve a settling duration of 0.123 s. The sliding mode controller-MPC improves performance with a settling time improvement of 0.072s. In simulations over 1 s with varying load torques of 5 N-m, and 10 N-m, the PID controller exhibits longer settling times compared to the SMC controller. For a 5 N-m step load at 1s, the PID settling time is 0.126s, while SMC is 0.061 s. For a 10 N-m step load at 1 s, PID is 0.074 s, and SMC is 0.038s. Figure 7 demonstrates that the SMC-MPC controller achieves the shortest settling times of 0.047 s and 0.02 s under load torques of 5 N-m and 10 N-m, respectively. This results in significantly smoother, quicker, and more stable performance over the conventional controllers, as detailed in Table 6.

#### Peak overshoot (M_p_)

Maximum overshoot is a crucial characteristic in EVs, influencing their performance and comfort. It refers to how much a system exceeds its target value during its transient response before stabilizing at the desired value^39,40^. The robustness of the control strategy is assessed using peak overshoot, which quantifies the maximum deviation in the speed response from the target value of 3000 rpm. Negative feedback can cause instability, leading to overshoot and torque oscillations. Equation (38) is used to calculate the percentage of peak overshoot.

38 $$\% M_p = \frac{|M_p - SS_{\mathrm{ref}}|}{SS_{\mathrm{ref}}} \times 100$$

where M_p_ represents the peak overshoot, while SS_ref_ denotes the steady-state reference speed. Figure 7 indicates that, under no-load conditions, the PID controller exhibits an overshoot of 26.03%, the SM controller shows an overshoot of 2.08%, and the SMC-MPC controller achieves a significantly lower overshoot of just 0.1966%. Under different torque levels of 5 N-m and 10 N-m, the PID controller’s overshoots are 24.96% and 22.91%, respectively. In contrast, the SM controller shows a moderate overshoot, while the SMC-MPC controller maintains the lowest overshoot, at 0.1316% and 0.0533% for 5 N-m and 10 N-m loads, respectively. This reduction substantially enhances the operational efficacy of the BLDC drive, as detailed in Table 6.

#### Steady state error (E_ss_)

The deviation between the desired and actual outputs after the transient responses have settled is known as steady-state error. For BLDC motors in EVs, this metric is crucial for assessing the effectiveness of torque voltage mitigation, impacting torque control, and overall performance^41^ Managing negative feedback to reduce transient overshoot while enhancing steady-state accuracy is key. Thus, understanding the balance between maximum overshoot and steady-state error is essential for optimizing BLDC motor controller design. The steady-state error percentage is determined using Eq. (39).

39 $$\% (E_{ss}) = \frac{\lvert SS_{\mathrm{actual}} - SS_{\mathrm{ref}} \rvert}{SS_{\mathrm{actual}}} \times 100$$

where %E_ss_ represents the steady-state error percentage, SS_ref_ is the reference value set at 3000 rpm, and SS_actual_ corresponds to the actual steady-state speed. The deviation between these values reflects the variation in speed. In this evaluation, the BLDC motor operating with no load demonstrates steady-state errors of 0.0832% with the PID controller and 0.068% with the SM controller. The SMC-MPC controller achieves the lowest error of 0.028%. Under torque levels of 5 N-m and 10 N-m, the PID controller’s errors.

are 0.0628% and 0.0564%, respectively, while the SM controller exhibits errors of 0.0432% and 0.0321%. As seen in Table 6, the SMC-MPC controller consistently maintains the smallest error of 0.001%, significantly enhancing system accuracy and stability.

### Motor performance analysis

**Fig. 8:**
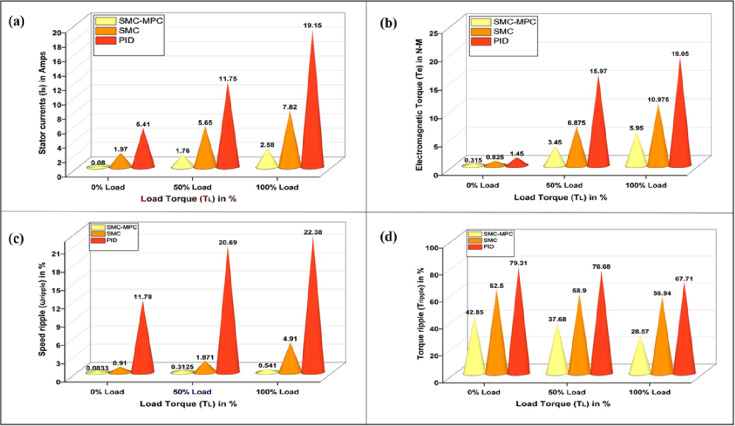
Comparison of motor performance analysis of various controllers at different load conditions (**a**) Stator current (**b**) Electromagnetic torque (**c**) Speed ripple (**d**) Torque ripple.

Evaluating and simulating BLDC motor operation is crucial for developing efficient drive systems in EVs, as it ensures precise speed control and addresses issues like harmonic distortion and torque pulsations. The main objective of this analysis is to evaluate the performance of various control strategies in managing torque fluctuations, focusing on key parameters such as phase current, torque output, and speed ripple, to assess their impact on motor behaviour and system stability as detailed in Table 7. The proposed controller is compared with the PID and SM controllers are shown in Fig. 8.

**Table 7:** Comparative study of motor performance analysis in SMC-MPC and conventional controllers.

Load torque (T_L_) state in (%)	Motor performance metrics	Different controller designs		
		PID	SMC	SMC-MPC
No Load torque (0%)	Stator current (Amps)	5.41	1.97	0.08
	Electromagnetic torque (N-m)	1.45	0.825	0.315
	Speed ripple ( r/s)	11.78	0.91	0.0833
	Torque ripple (%)	79.31	62.5	42.85
Medium Load torque (50%)	Stator current (Amps)	11.75	5.65	1.76
	Electromagnetic torque (N-m)	15.97	6.875	3.45
	Speed ripple ( r/s)	20.69	1.871	0.3125
	Torque ripple (%)	76.68	58.9	37.68
Maximum Load torque (100%)	Stator current (Amps)	19.15	7.82	2.58
	Electromagnetic torque (N-m)	19.05	10.975	5.95
	Speed ripple ( r/s)	22.38	4.91	0.541
	Torque ripple (%)	67.71	56.94	28.57

#### Stator current (I_s_)

**Fig. 9:**
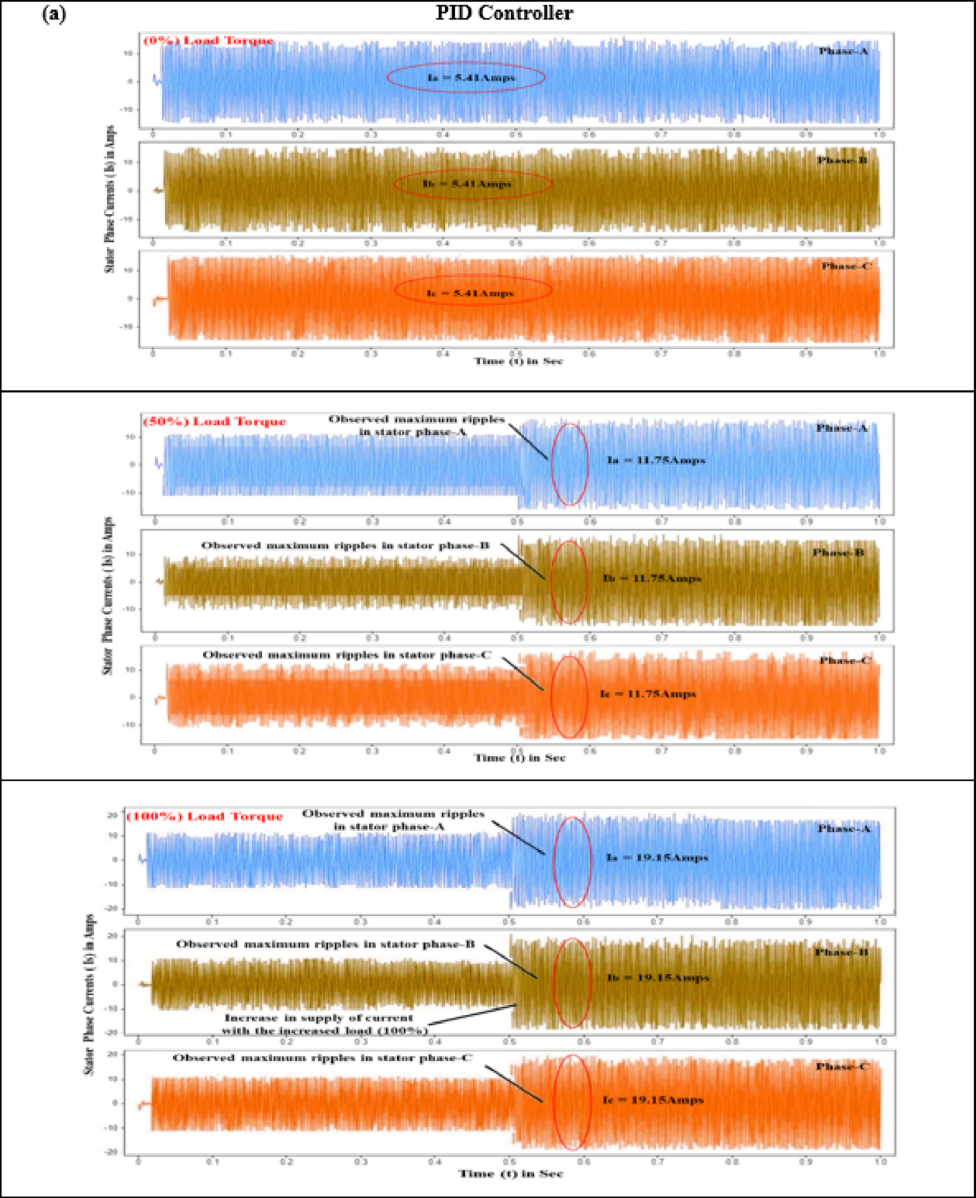

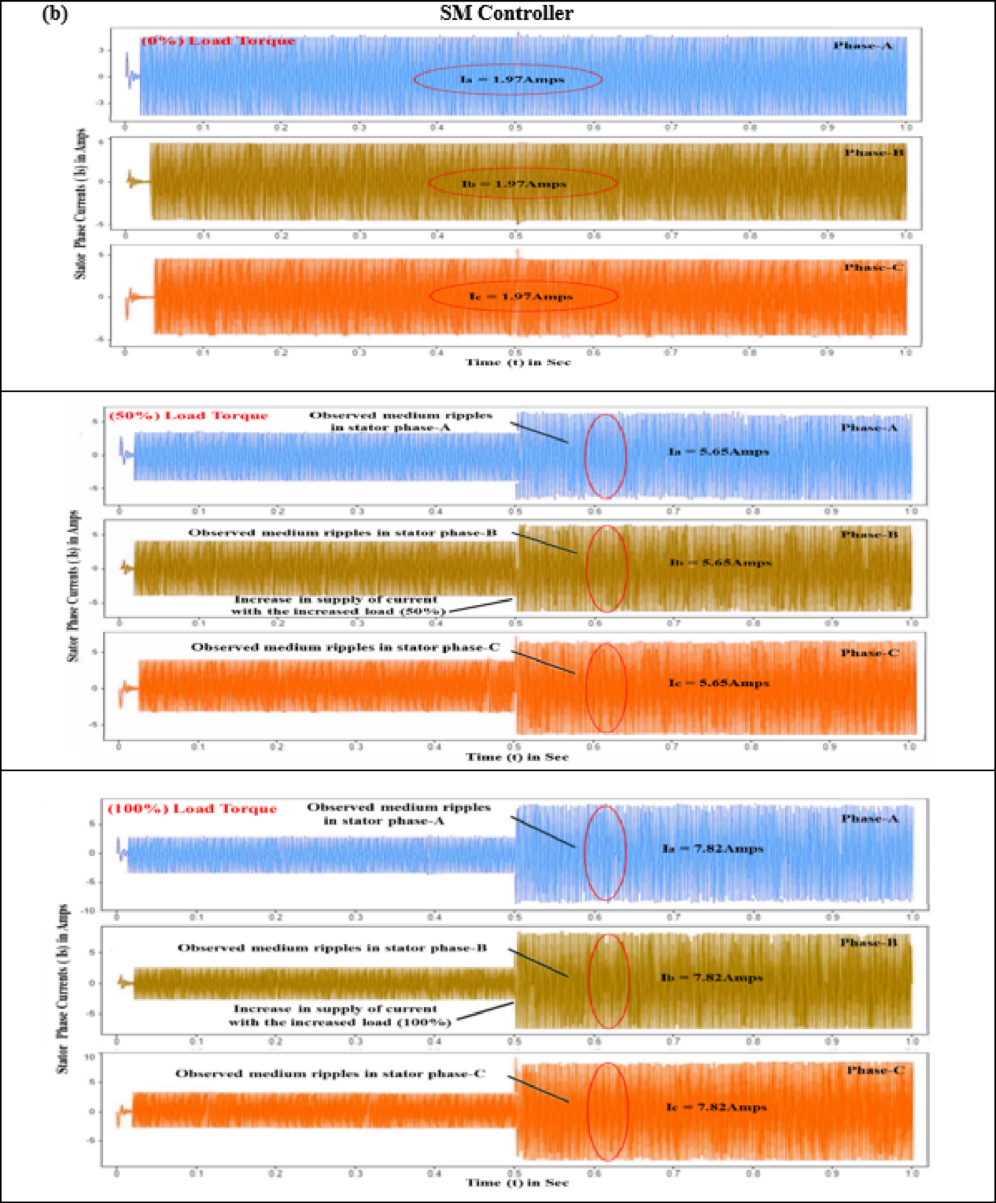

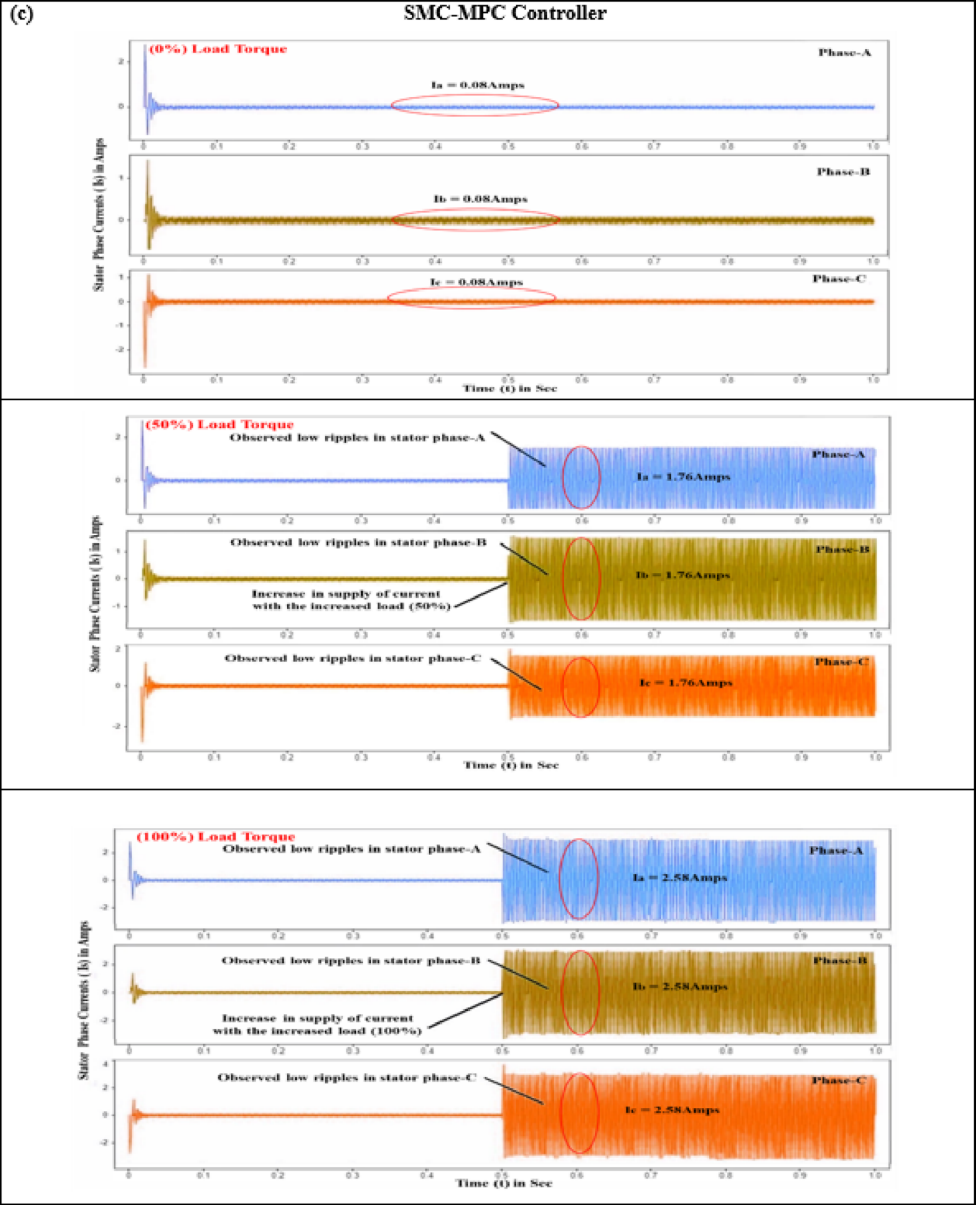
Stator current response of BLDC motor at different load conditions (**a**) PID Controller. (**b**) SM controller. (**c**) SMC-MPC controller.

Precise stator current control is crucial for minimizing periodic torque component, improving motor drive charcteristics, and ensuring smooth and quiet operation in EVs. This is achieved using sophisticated electronic control units with high-speed processors and advanced algorithms. The three-phase stator currents, i_a_, i_b_, and i_c_, each separated by a 120-degree electrical phase shift, are essential for ensuring stable and efficient power drive performance. Evaluations of controller performance under torque loads varying from 0 to 10 N-m reveal that conventional controllers exhibit erratic speed variations, primarily attributed to significant ripple effects in the stator phase currents (a, b, and c). Specifically, when a BLDC motor starts with no load torque, it experiences significant deviations and ripples in the stator current. PID and SMC controllers exhibit current ripples of 5.41 A and 1.97 A, respectively, while the proposed controller reduces this to a mere 0.08 A, as seen in Fig. 9.

Additional analysis with load torques of 5 N·m and 10 N·m reveals that the PID controller produces stator phase currents of 11.75 A and 19.15 A. In comparison, the SM controller generates currents of 5.65 A and 7.82 A under the same conditions. As demonstrated in Table 7, the SMC-MPC controller notably reduces stator currents to 1.76 A and 2.58 A, significantly lowering fluctuations and enhancing performance.

#### Electromagnetic torque (T_e_)

The interaction of magnetic fields between the stator and rotor in BLDC motors, known as electromagnetic torque, leads to torque variation during phase commutation. This phenomenon results in noise, vibrations, and reduced efficiency, with high-frequency fluctuations exacerbating the issue and necessitating advanced control strategies. Effective control strategies must optimize torque while minimizing ripple to ensure smooth and quiet operation. The performance of electromagnetic torque is contingent upon the motor’s energy capacity and power delivery. At the start of the test with a zero N·m torque load level, the PID, SMC, and SMC-MPC controllers produce torque pulsations of 1.45 N·m, 0.825 N·m, and 0.315 N·m. During this initial stage, the responses of current and torque ripple are adversely affected. Integrating an adaptive learning algorithm based on ANN-Fuzzy with the SMC-MPC controller significantly enhances torque, stator current, and speed, while concurrently minimizing deviations to a minimal level. When subjected to step inputs of 5 N·m and 10 N·m, the PID and SMC controllers achieve peak torques of 15.97 N·m and 19.05 N·m, and 6.875 N·m and 10.975 N·m, respectively. As outlined in Table 7, the SMC-MPC controller achieves significantly lower maximum torques of 3.45 N·m and 5.95 N·m, effectively reducing the efficiency periodic torque component and enhancing operational performance.

#### Speed ripple (ω_ripple_)

Characterized by periodic fluctuations in torque, torque distortion is closely associated with speed ripple, which reflects changes in the motor’s rotational speed. Mitigating speed ripple aids in reducing cyclic torque deviation, thereby enhancing the efficiency of BLDC motor drives. The imbalance between motor and load torque induces speed ripple, exacerbating vibration and noise. Rapid fluctuations in rotor speed are influenced by current ripples in the non-commutating phase^42,43^. By managing phase current fluctuations and utilizing diverse control approaches, speed ripple can be effectively minimized. The percentage of speed ripple is determined using the following Eqs. (40),

40 $$\:{\%\:(\omega\:}_{ripple})=\frac{{\omega\:}_{max}-{\omega\:}_{min}}{{\omega\:}_{max}+{\omega\:}_{min}}*100$$

where % ω_ripple_ denotes the percentage of speed ripple in the rotor speed of the BLDC motor.

The highest and lowest speed values ω_max_ and ω_min_ are recorded at a rotational speed of 3000 rpm. Figure 7 illustrates that the proposed SMC-MPC controller attains a speed ripple of 0.0833 r/s. In contrast, the PID and SMC controllers produce ripples of 11.78 and 0.91 r/s, respectively, during a 0 to 1 s simulation at 3000 rpm with no load applied. Under sudden load changes with torques of 5 N-m and 10 N-m throughout 1s, the speed ripples are 20.69 r/s, 1.871 r/s, and 0.3125 r/s for the three control schemes, increasing to 22.38 r/s, 4.91 r/s, and 0.541 r/s for higher torque values summarized in Table 6.

**Fig. 10:**
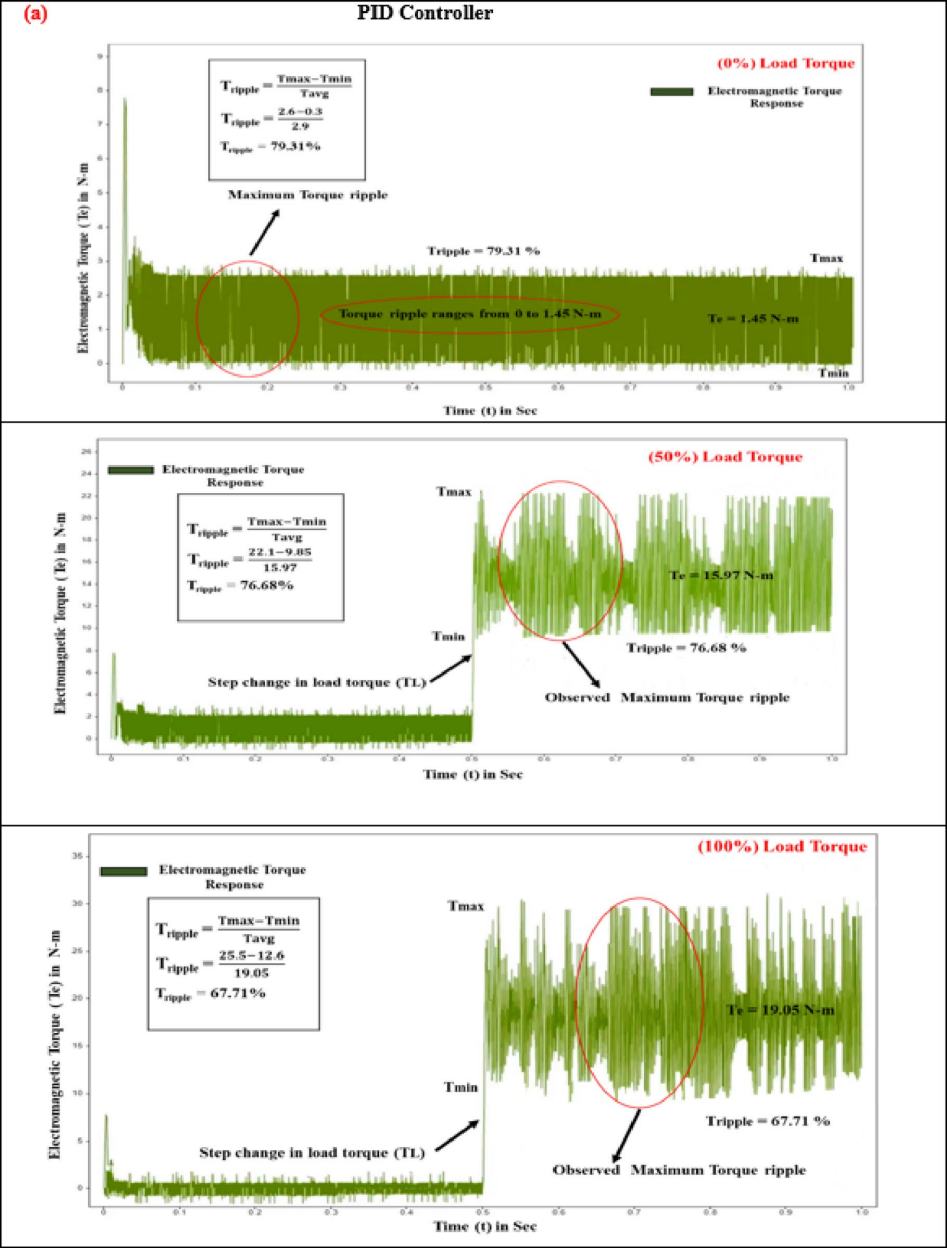

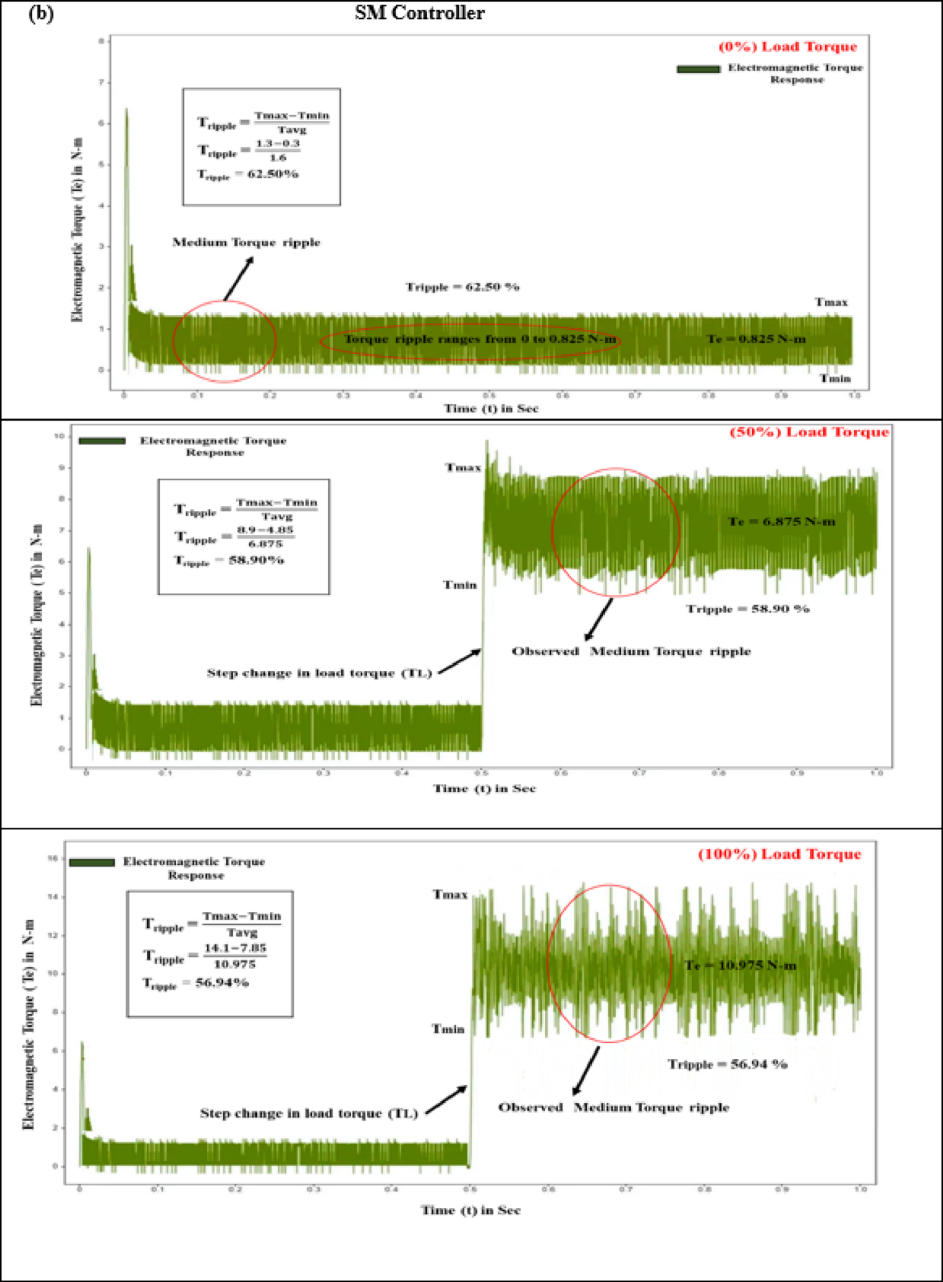

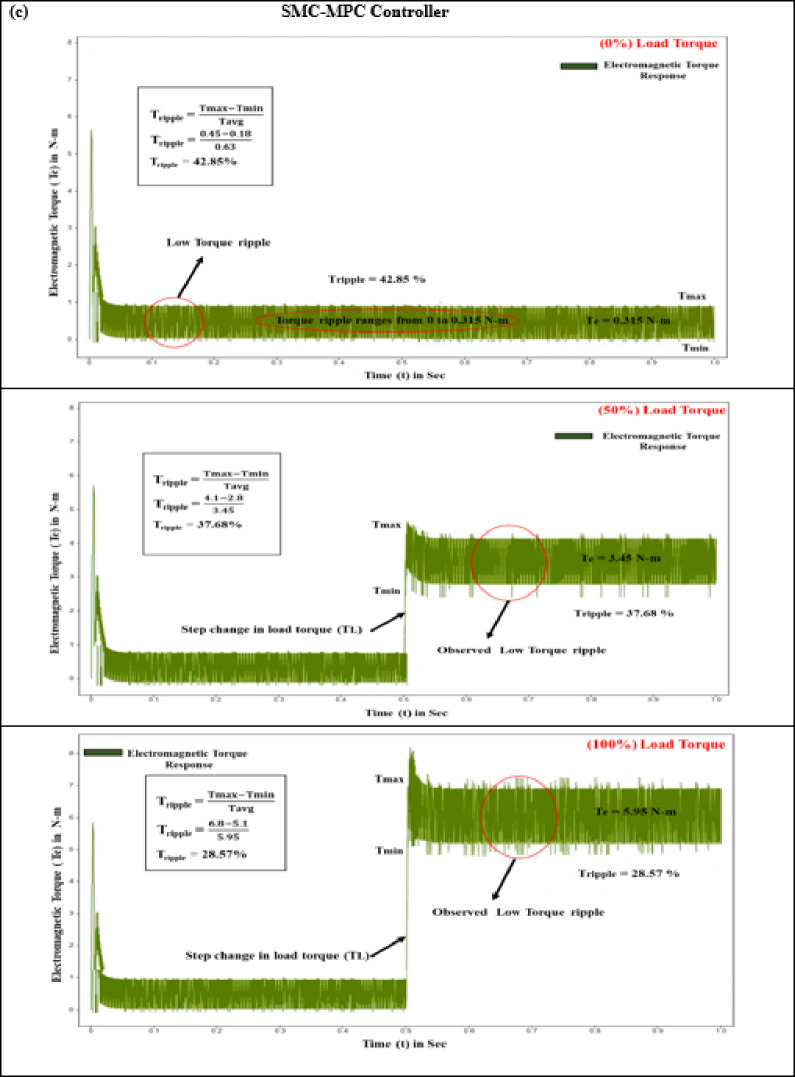
Electromagnetic torque response of BLDC motor at different load conditions (**a**) PID Controller. (**b**) SM controller. (**c**) SMC-MPC controller.

#### Torque ripple (T_ripple_)

Analyzing torque variations is essential for optimizing BLDC motor drive charcteristics in EVs, as it impacts motion control, efficiency, and the overall driving experience. This ripple, arising from the interactions of magnetic fields between the stator and rotor, causes fluctuations in torque output across both low and high-speed operations. By understanding the motor’s architecture, design parameters, and control factors, and applying effective control strategies, the steady-state and dynamic enhancability of EV drive systems are enhanced. This results in reduced noise and vibration while improving overall performance^44–47^. Equation (41) is used to calculate the percentage of ripple present in the torque.

41 $${\mathrm{\%}\:(\mathrm{T}}_{\mathrm{r}\mathrm{i}\mathrm{p}\mathrm{p}\mathrm{l}\mathrm{e}})=\frac{{\mathrm{T}}_{\mathrm{m}\mathrm{a}\mathrm{x}}-{\mathrm{T}}_{\mathrm{m}\mathrm{i}\mathrm{n}}}{{\mathrm{T}}_{\mathrm{a}\mathrm{v}\mathrm{g}}}\mathrm{*}100$$

where % T_ripple_ represents the percentage of torque variation in the BLDC motor’s rotational speed.

During zero-load conditions across a 0 to 1 s period, the proposed SMC-MPC controller achieves a torque ripple of 42.85%, which is significantly lower than the 79.31% and 62.5% observed with the PID and SM controllers, respectively. When operating a BLDC motor at 3000 rpm with a load increasing from 5 N-m to 10 N-m, the SMC-MPC controller reduces the Periodic torque component to approximately 28.57%, compared to 67.71% and 56.94% for PID and SM controllers, respectively. These results, shown in Fig. 10; Table 7, highlight that the self-learning-based Sliding mode controller provides superior torque response and markedly improved driving performance in electric vehicles.

## Development of BLDC motor controllers using HIL simulation

The framework of this study is predicated on a Hardware-in-the-Loop methodology, wherein torque ripple mitigation control strategies are initially modeled and simulated within a MATLAB/Simulink environment. This simulation platform facilitates rigorous testing and iterative refinement of advanced control algorithms, including PID, sliding mode control, and the advanced integration of sliding mode control with model predictive control. Upon achieving optimal performance in the simulation phase, these control algorithms are subsequently implemented in real-time hardware experimentation, leveraging a 3 kW BLDC motor setup that is emblematic of standard EV applications.

### Experimental design for comparative analysis of BLDC motor controllers

**Fig. 11:**
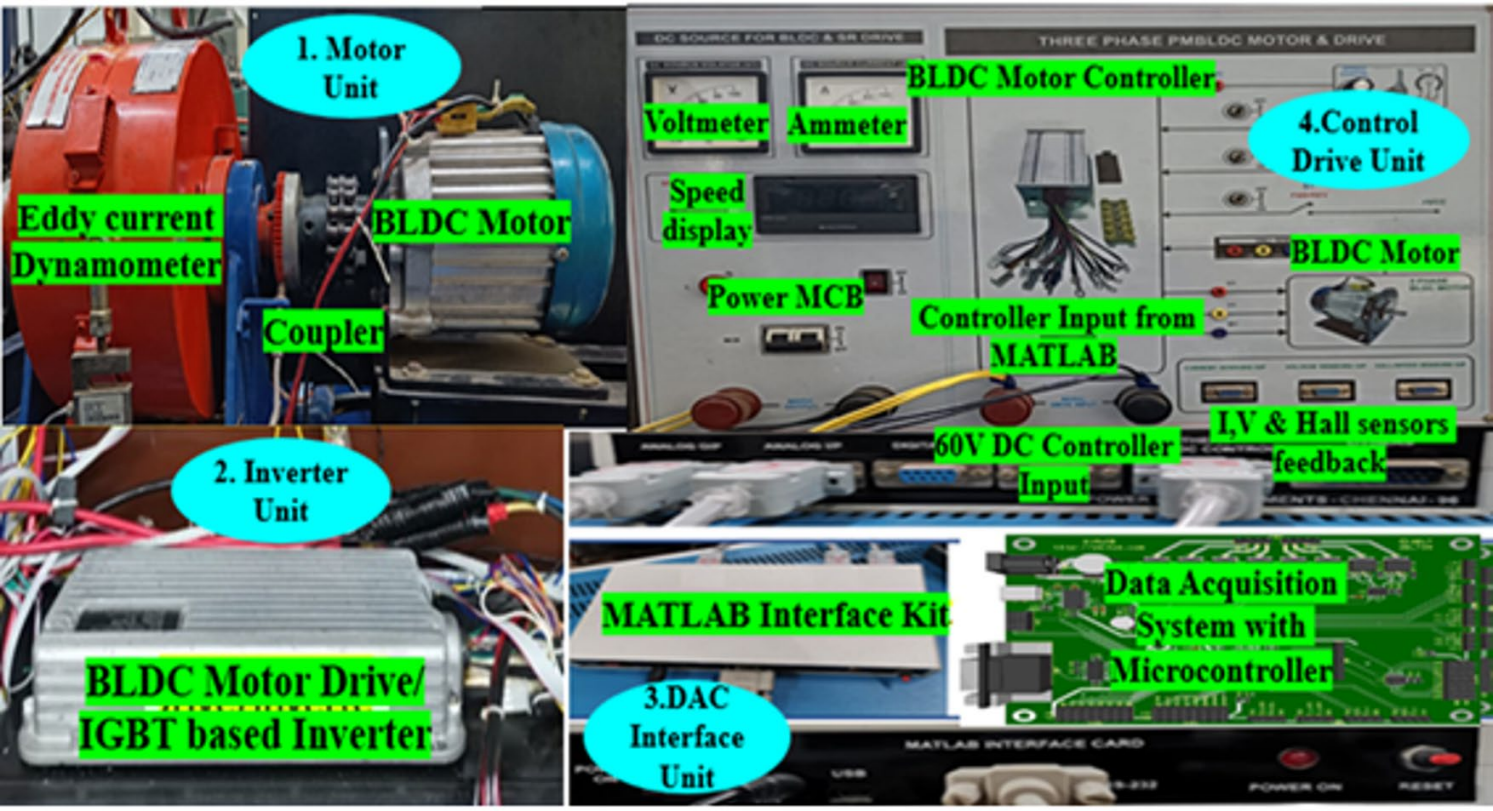
Hardware of experimental setup arrangement for BLDC motor.

As illustrated in Fig. 11, the real-time experimental hardware setup comprises a computer system, a power supply module, a MATLAB control interface, a data acquisition system with an embedded microcontroller, and a three-phase brushless DC motor. Additionally, it is paired with a 7.5 kW water-cooled eddy current brake to enable accurate load simulation. MATLAB/Simulink simulations are validated via a USB-connected DAQ, while an RS-232 connection facilitates real-time communication between the embedded controller and the motor drive. The dynamometer simulates loads from 0 to 10 Nm, and the system handles speed signals from 0 to 5 V and up to 3000 rpm. Performance is analyzed using PID, SMC, and SMC-MPC strategies to evaluate the undesired torque component and efficiency. The proposed SM-MPC dual-loop control with a self-learning approach for torque irregularity reduction in a BLDC motor used in two-wheeler EVs is validated by the experimental setup. It comprises four main units:

- Motor Unit: A 3 kW, 60 V, 3000 rpm BLDC motor coupled with an eddy current dynamometer for load and torque analysis. Key parameters: 65 A current, 10 Nm torque, 6 poles, 2.875 Ω resistance, 0.0085 H inductance, 1 mm air gap, 0° rotor position.
- Inverter Unit: A two-pulse IGBT-based VSI (60 V input, 15 kHz switching) controlled by an STM32 microcontroller, implementing SMC for speed and MPC for current.
- DAC & DAQ Unit: A MATLAB interface kit with a microcontroller-based DAC ensures real-time data exchange and captures current, voltage, speed and torque feedback.
- Control Drive Unit: Includes controller, sensors and displays. MATLAB’s MBC Toolbox with I-optimal DOE fine-tunes control parameters.

The setup operates in MIL, SIL and HIL environments, ensuring realistic validation, dynamic performance improvement, and torque ripple minimization.

### Mapping-based efficiency analysis of motor control strategies

The experimental results demonstrate a substantial decrease in torque oscillation when using the SMC-MPC controller, relative to conventional PID and SMC methods. The transition from simulation to hardware validation underscores the robustness of the rapid prototyping methodology, with performance gains observed in the simulation phase consistently replicated in hardware experiments. This approach effectively investigates and validates transient simulations of electrical systems, replicates testing results, and identifies potential issues before real-time deployment. Additionally, the torque-speed efficiency chart generated from the hardware tests confirms the superior energy utilization of the optimized controllers, further validating their applicability in EV systems.

The practicality of the proposed SMC-MPC controller in alleviating torque voltage in BLDC motors is validated by comparing its performance with that of conventional controllers, employing real-time Hardware-in-the-Loop simulations. Table 8 delineates the torque ripple results for each controller, ascertained through both experimental and simulation approaches. The experimental results demonstrate a high degree of congruence with simulation results, as long as a uniform switching frequency is sustained across different load conditions. The interplay between inverter switching frequency and torque fluctuation is integral to assessing the efficacy of the proposed sliding mode controller-model predictive controller approach for BLDC motors. An elevation in switching frequency typically mitigates fluctuations present in the torque by enabling more granular current modulation; however, excessively high frequencies may induce elevated switching losses and compromise overall system efficiency. The SMC-MPC controller adeptly navigates this balance by leveraging sliding mode control to fortify robustness against system perturbations and employing model predictive control for dynamic modulation of the switching frequency. This dual-faceted optimization ensures a reduction in torque voltage while maintaining operational efficacy. Empirical validation reveals that the SMC-MPC controller markedly exceeds traditional methodologies in attenuating fluctuations in the ripple across a spectrum of switching frequencies, thereby substantiating its efficacy in enhancing motor drive performance and efficiency for electric vehicles.

**Table 8:** Simulated and experimental validation of torque ripple in BLDC motors.

Validation metrics	Load torque (T_L_) condition in %	Various controllers		
		PID	SMC	Proposed SMC-MPC
Torque ripple (T_ripple_) in % (simulation)	No load torque (0%)	79.31	62.50	42.85
	Medium load torque (50%)	76.68	58.90	37.68
	Max load torque (100%)	67.71	56.94	28.57
Torque ripple (T_ripple_) in % (experimentation)	No load torque (0%)	79.94	63.21	43.68
	Medium load torque (50%)	77.03	59.61	38.02
	Max load torque (100%)	68.26	56.97	28.98

The superior torque smoothness and dynamic response of the proposed ANN-FLC-SMC-MPC controller stem from coordinated regulation of torque, flux, and current. The inner MPC loop predicts future motor states and applies optimal voltage vectors to maintain current–back-EMF alignment, thereby minimising torque fluctuations and accelerating the transient response. The outer SMC ensures robust speed tracking by enforcing instantaneous torque compensation against load disturbances, while MPC smoothing suppresses chattering-induced current oscillations. The adaptive ANN-FLC layer dynamically tunes control gains to preserve torque linearity and flux stability under parameter and temperature variations, thereby reducing current asymmetry and electromagnetic vibration. This hybrid integration enables smoother commutation, reduced flux fluctuations, and stable electromagnetic energy conversion. Quantitatively, torque ripple decreased from 73.2% (PID) and 48.5% (SMC) to 36.7% with SMC-MPC, and further to 28.98% with ANN-FLC, confirming improved flux–current coordination and uniform torque generation.

**Fig. 12:**
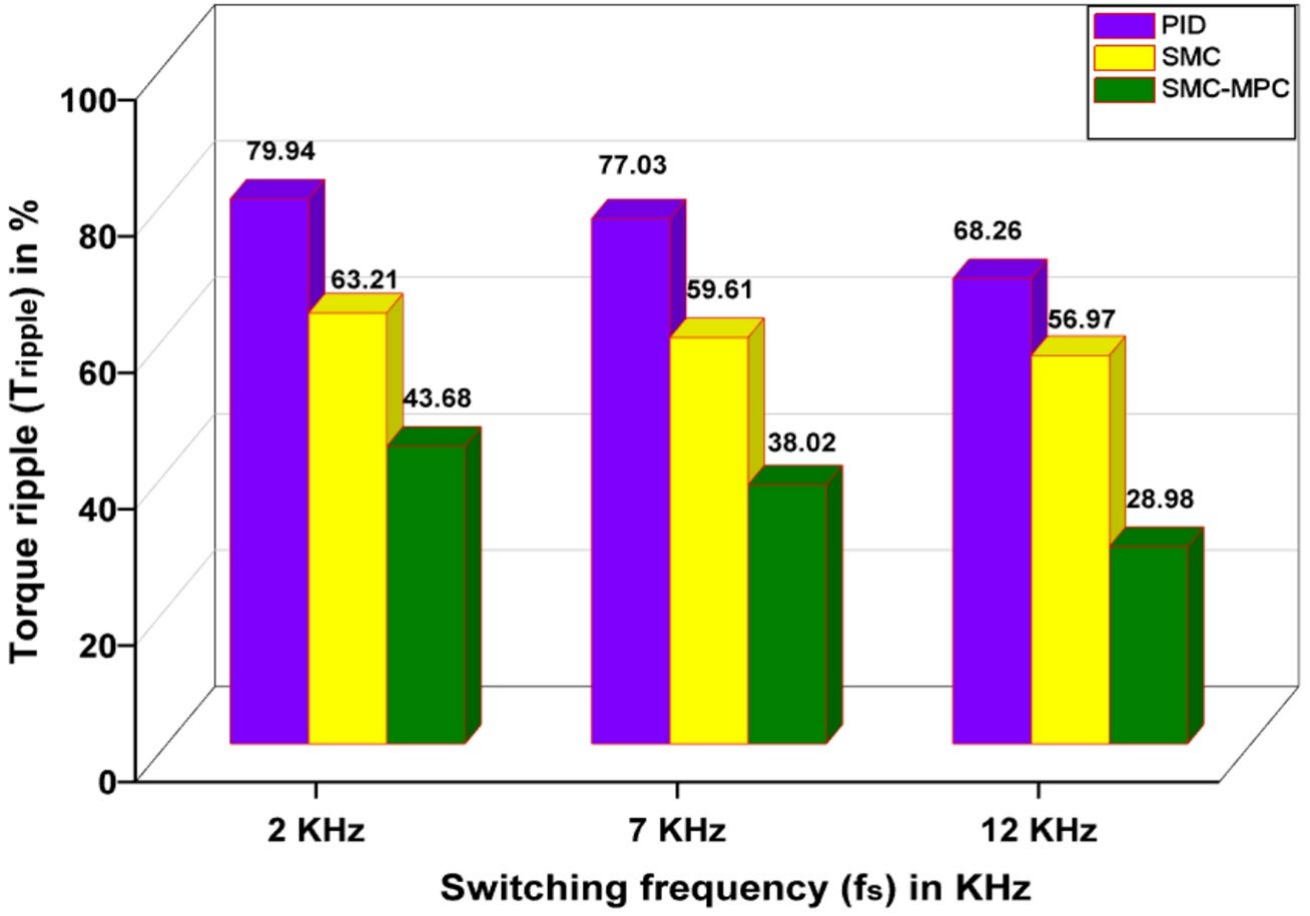
Experiment torque ripple analysis of BLDC motor with Various Controllers under the same switching frequency.

The inverter operates at a switching frequency of 12.5 kHz, achieving an optimal compromise between reducing switching losses and sustaining effective motor control. Equation (42) calculates the amplitude of the inverter switching frequency,

42 $$\:fs=\frac{{\mathrm{N}}_{\mathrm{T}}}{6\mathrm{T}}$$

where, N_T_ denotes the time-averaged switching frequency across a duration T, and fs​ denotes the frequency of switching events. Figure 12 illustrates experimental data depicting torque fluctuations in a BLDC motor under various controllers at a constant switching frequency.

**Fig. 13:**
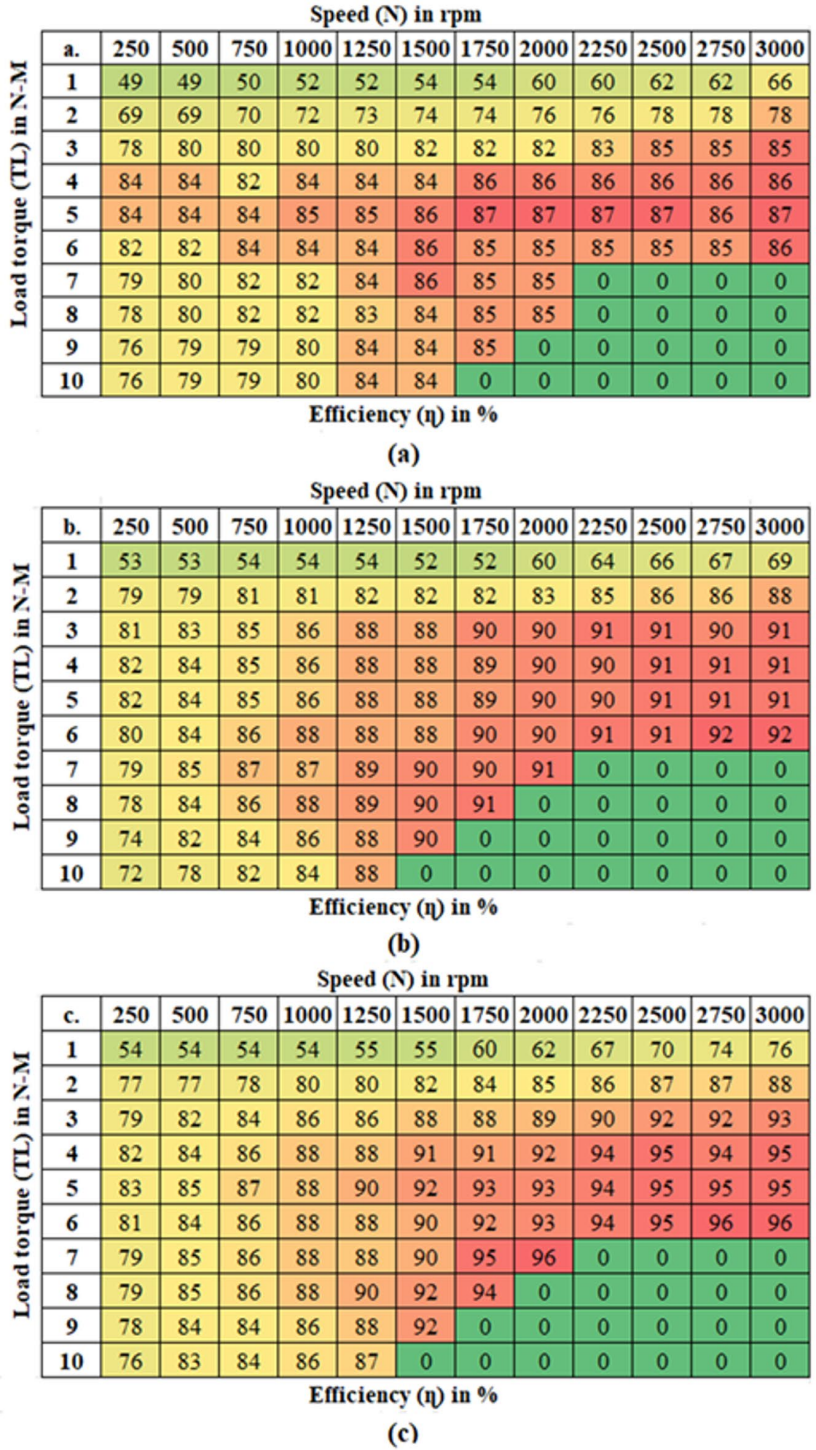
Efficiency map development of various controllers for BLDC motor in EVs. (**a**) PID controller. (**b**) Sliding mode controller. (**c**) Sliding mode-model predictive controller.

The analysis reveals that the proposed SMC controller markedly mitigates undesired torque component, especially as the switching frequency is increased across different load torque levels^48^. A detailed Motor efficiency contour for the BLDC motor has been meticulously developed to significantly improve torque oscillation reduction, providing an in-depth analysis of controller validation outcomes across various providing an exhaustive depiction of the motor drive’s performance across the entire range of operational conditions^49,50^ as load and speed level conditions. This motor performance map illustrates the peak achievable efficiency for any speed-torque relationship, depicted in Fig. 13.

Based on the validation, the developed SMC-MPC controller shows enhanced performance compared to traditional control methods, as illustrated by the mapping of efficiency. Table 9 details that the maximum quantified efficiencies achieved by the various controllers are 86.38%, 92.43%, and 96.47%, respectively.

**Table 9:** Comparative quantified efficiency evaluation of the proposed SMC-MPC controller with conventional controllers.

Load torque (T_L_) in (%)	Various controllers		
	PID	SMC	Proposed SMC-MPC
No load torque (0%)	66.5	70.8	74.2
Medium load torque (50%)	84.0	87.8	91.6
Maximum load torque (100%)	85.3	89.1	94.0
Overall average	78.6	82.6	86.6
Peak efficiency observed	86.38	92.43	96.47

In light of the torque ripple and efficiency analysis in the experiment, it turns out that the proposed SMC-MPC controller provides outstanding performance in both torque and speed regulation of BLDC motors in EV applications, as described in Table 10.

**Table 10:** Summary of the comparative controller performance results.

Load torque (T_L_) in (%)	Overall performance parameters	Controller performance evaluation		
		PID	SMC	SMC-MPC
No load torque (0%)	Rise time (s)	0.079	0.042	0.02
	Settling time (s)	0.215	0.123	0.072
	Peak overshoot (%)	26.03	2.08	0.1966
	Steady-state error (%)	0.0832	0.068	0.028
	Torque ripple (%)	79.31	62.5	42.85
	Efficiency (%)	66.5	70.8	74.2
Medium load torque (50%)	Rise time (s)	0.062	0.034	0.02
	Settling time (s)	0.126	0.061	0.047
	Peak overshoot (%)	24.96	1.916	0.1316
	Steady-state error (%)	0.0628	0.0432	0.0025
	Torque ripple (%)	76.68	58.9	37.68
	Efficiency (%)	84.0	87.8	91.6
Maximum load torque (100%)	Rise time (s)	0.057	0.029	0.01
	Settling time (s)	0.074	0.038	0.02
	Peak overshoot (%)	22.91	1.106	0.0533
	Steady-state error (%)	0.0564	0.0321	0.001
	Torque ripple (%)	67.71	56.94	28.57
	Efficiency (%)	86.38	92.43	96.47
Overall performance in EV		Low	Medium	High

## Conclusions

This study expounds upon the formulation of a dual-loop sliding mode model predictive controller strategy, intricately engineered for BLDC motors within EVs. The primary objective of this advanced control framework is to minimise torque variation while attaining superior speed stabilization. The strategy synergistically integrates the predictive capabilities of Model Predictive Control with the inherent robustness of Sliding Mode Control, facilitated by a dual-loop architecture comprising an inner current loop and an outer speed loop. This configuration ensures rigorous stator current regulation, thereby optimizing overall motor efficiency. Extensive MATLAB/SIMULINK model analyses, conducted under varying load torque levels of 0%, 50% and 100% while maintaining a constant rotational speed of 3000 rpm, highlight the significant superiority of the SMC-MPC controller over conventional PID and SM controllers. The SMC-MPC model, augmented by an advanced ANN-Fuzzy self-learning algorithm, exhibits substantial enhancements in time-domain response metrics, achieving prompt stabilization within 0.02 s, with an exceptionally reduced rise time (0.01s), slight maximum overshoot (0.0533%) and insignificant steady-state error (0.001%). Further, the SMC-MPC controller significantly enhances key motor performance metrics, including electromagnetic torque, stator current, speed fluctuation, and torque ripple with 28.57% reduction, thereby surpassing the effectiveness of existing control strategies. The efficacy of the SMC-MPC approach is corroborated through rigorous Hardware-in-the-Loop (HIL) testing, which reveals a peak efficiency of 96.47% across different load conditions. This investigation establishes the sliding mode model predictive controller as a robust, efficient and highly reliable control strategy, rendering it a compelling solution for advanced EV applications.

Despite these numerical results, this study presents a hybrid SMC–MPC–ANN–FLC framework for torque ripple mitigation in BLDC motors, validated through Software-in-the-Loop (SIL) simulation. By assuring smoother torque delivery, lower NVH, and less mechanical stress on gearbox components, the observed torque ripple reduction (from changes 43.68 to 28.98%) directly improves EV drivetrains, increasing durability and reducing maintenance requirements. Extended driving range and increased energy efficiency are two benefits of improved current control, which also reduces high-frequency losses. Moreover, SIL-based timing and computational analyses confirm the controller’s feasibility for real-time embedded implementation. An important step towards full-scale deployment is the planned future hardware-in-the-loop (HIL) and prototype validations, which will evaluate thermal loading, dependability and overall efficiency under realistic electric vehicle (EV) operating conditions.

The salient features of the proposed SMC-MPC controller technique are as follows:

- Exhibits superior efficiency and lower speed ripple than conventional methods at the same switching frequency.
- Expedited time-domain response and effective speed stabilization with minimum torque oscillation over a wide range.
- Enhanced robustness to system dynamic changes in parameters and uncertainties.

## Data Availability

All data generated or analyzed during this study are included in this published article. Additional information is available from the corresponding author upon reasonable request.

## Abbreviations

ANN: Artificial neural network
BLDC: Brushless direct current
E_b_: Back EMF
E_ss_: Steady-state error
EV: Electric vehicle
HIL: Hardware-in-the-loop
MIL: Model-in-the-loop
M_P_: Peak overshoot
MPC: Model predictive controller
PID: Proportional integral derivative
SMC: Sliding mode controller
T_e_: Electromagnetic torque
T_ripple_: Torque ripple
ω_ripple_: Speed ripple
